# Monoallelic and bi-allelic variants in *NCDN* cause neurodevelopmental delay, intellectual disability, and epilepsy

**DOI:** 10.1016/j.ajhg.2021.02.015

**Published:** 2021-03-11

**Authors:** Ambrin Fatima, Jan Hoeber, Jens Schuster, Eriko Koshimizu, Carolina Maya-Gonzalez, Boris Keren, Cyril Mignot, Talia Akram, Zafar Ali, Satoko Miyatake, Junpei Tanigawa, Takayoshi Koike, Mitsuhiro Kato, Yoshiko Murakami, Uzma Abdullah, Muhammad Akhtar Ali, Rein Fadoul, Loora Laan, Casimiro Castillejo-López, Maarika Liik, Zhe Jin, Bryndis Birnir, Naomichi Matsumoto, Shahid M. Baig, Joakim Klar, Niklas Dahl

**Affiliations:** 1 Department of Immunology, Genetics and Pathology, Uppsala University and Science for Life Laboratory, Box 815, 751 08 Uppsala, Sweden; 2 Department of Human Genetics, Yokohama City University Graduate School of Medicine, Yokohama, Kanagawa 236-0004, Japan; 3 Center for Molecular and Chromosomal Genetics APHP, Sorbonne University, Pitié-Salpêtrière Hospital, 47-83 Boulevard de l’Hôpital, 75013 Paris, France; 4 Human Molecular Genetics Laboratory, National Institute for Biotechnology and Genetic Engineering, Faisalabad 38000, Pakistan; 5 Clinical Genetics Department, Yokohama City University Hospital, Yokohama, Kanagawa 236-0004, Japan; 6 Department of Pediatrics, Osaka University Graduate School of Medicine, Osaka 565-0871, Japan; 7 National Epilepsy Center, NHO Shizuoka Institute of Epilepsy and Neurological Disorders, Shizuoka 420-8688, Japan; 8 Department of Pediatrics, Showa University School of Medicine, Tokyo 142-8666, Japan; 9 Research Institute for Microbial Diseases, Osaka University, Osaka 565-0871, Japan; 10 University Institute of Biochemistry and Biotechnology, PMAS-Arid Agriculture University, Rawalpindi 46301, Pakistan; 11 School of Biological Sciences, University of the Punjab, Lahore 54590, Pakistan; 12 Unit of Clinical Neurophysiology, Uppsala University Hospital, 751 85 Uppsala, Sweden; 13 Department of Medical Cell Biology, Biomedical Centre, Uppsala University, 751 08 Uppsala, Sweden

**Keywords:** *NCDN* gene, neurodevelopmental delay, intellectual disability, epilepsy, speech delay, neurite formation, mGluR5 signaling, missense variant, *de novo* variant

## Abstract

Neurochondrin (NCDN) is a cytoplasmatic neural protein of importance for neural growth, glutamate receptor (mGluR) signaling, and synaptic plasticity. Conditional loss of *Ncdn* in mice neural tissue causes depressive-like behaviors, impaired spatial learning, and epileptic seizures. We report on *NCDN* missense variants in six affected individuals with variable degrees of developmental delay, intellectual disability (ID), and seizures. Three siblings were found homozygous for a *NCDN* missense variant, whereas another three unrelated individuals carried different *de novo* missense variants in *NCDN.* We assayed the missense variants for their capability to rescue impaired neurite formation in human neuroblastoma (SH-SY5Y) cells depleted of NCDN. Overexpression of wild-type *NCDN* rescued the neurite-phenotype in contrast to expression of *NCDN* containing the variants of affected individuals. Two missense variants, associated with severe neurodevelopmental features and epilepsy, were unable to restore mGluR5-induced ERK phosphorylation. Electrophysiological analysis of SH-SY5Y cells depleted of NCDN exhibited altered membrane potential and impaired action potentials at repolarization, suggesting NCDN to be required for normal biophysical properties. Using available transcriptome data from human fetal cortex, we show that *NCDN* is highly expressed in maturing excitatory neurons. In combination, our data provide evidence that bi-allelic and *de novo* variants in *NCDN* cause a clinically variable form of neurodevelopmental delay and epilepsy, highlighting a critical role for NCDN in human brain development.

## Main text

Neurodevelopmental disorders (NDDs) are a clinically heterogeneous group of cognitive, neurological, and neuropsychiatric conditions that manifest during childhood.^1^ Although the major fraction of NDDs has a multifactorial origin, identification of the genetic basis for rare Mendelian forms has brought important insights into molecular pathways and functional modules of importance for the formation and homeostasis of the human brain.^2^ Furthermore, understanding the precise cause of Mendelian NDDs provides critical information for diagnosis, counselling, and the development of specific treatment options.^3^ With the implementation of next-generation sequencing technologies, gene variants in hundreds of genes have been associated with Mendelian NDDs, illustrating an extensive genetic heterogeneity.^4^^,^^5^ It is now estimated that almost half of all affected individuals with severe NDD have a pathogenic and protein-coding *de novo* variant,^4^ whereas inherited and recessive acting variants account for only a small fraction of resolved and affected individuals in outbred populations.^6^ Despite the enormous progress over the last decades, it is now generally accepted that many genes and genetic mechanisms associated with neurodevelopmental delay remain to be identified in efforts to improve the diagnostic yield.^7^^,^^8^

We report on bi-allelic and *de novo* variants in *NCDN* (MIM: 608458) causing NDD and epilepsy with broad clinical variability. The *NCDN* gene encodes neurochondrin (NCDN), initially named norbin, a leucine-rich cytoplasmic protein widely expressed in developing and adult brain.^9^^,^^10^ The protein is highly conserved and enriched in α helices but without homologies to known functional domains.^11^ Originally identified as a regulator of neurite outgrowth,^12^ NCDN is an adaptor protein for G-coupled receptors^13^ and modulates metabotropic glutamate receptor 5 (mGluR5) signaling of importance for synaptic plasticity in the cerebellum and hippocampus.^14^ Mice with conditional depletion of *Ncdn* in the nervous system exhibit epileptic seizures and show spatial learning defects.^15^ Furthermore, *Ncdn*−/− mice show impaired neural proliferation and maturation, reduced amounts of mGluR5 on the surface of cortical neurons, and abolished long-term potentiation (LTP),^14^^,^^16^ a form of neuronal plasticity of importance for learning and memory.^16^

The study was conducted on four families and performed in accordance with the Declaration of Helsinki and after written informed consent from all legal guardians. The study was approved by the regional ethical committee of Uppsala, Sweden; the ethical review board at National Institute for Biotechnology and Genetic Engineering, Faisalabad; and the Committees for Ethical Issues at Showa University School of Medicine, Osaka University Graduate School of Medicine, and Yokohama City University School of Medicine.

**Figure 1:**
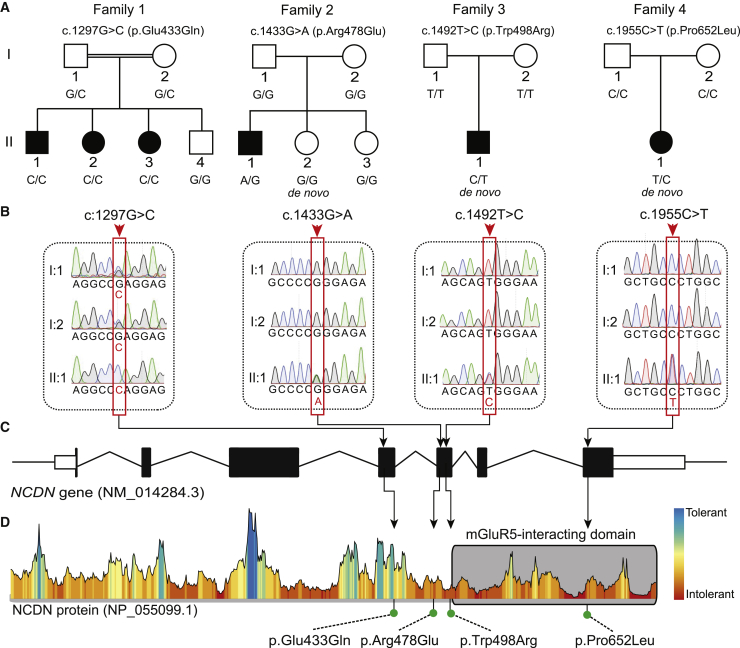
Segregation of rare *NCDN* missense variants in families with neurodevelopmental phenotypes (A) Pedigrees of four unrelated families with affected members indicated as filled circles (females) and squares (males). Double horizontal lines indicate consanguinity (first cousins). (B) Representative chromatograms are shown for each *NCDN* variant indicated by vertical red boxes and arrowheads. (C) Schematic representation of *NCDN* spanning seven exons with relative positions of variants identified in this study. Filled boxed regions denote coding sequences and white boxed regions denote untranslated regions. (D) Tolerance landscape visualization for missense variants in NCDN via MetaDome^20^ with relative positions of the four predicted amino acid (aa) substitutions. The graph indicates that variants close to, or within, the mGluR5-interacting domain (gray box)^14^ are less tolerated. The p.Glu433 position, affected by the recessive missense variant c.1297G>C, is “slightly tolerant,” while the three heterozygous *de novo* variants are “intolerant” for aa substitutions.

We initially identified a consanguineous family of Pakistani origin segregating three siblings presenting with similar degree of neurodevelopmental delay, mild ID, and seizures. The healthy parents are first cousins and without family history of NDD. Whole-exome sequencing (WES) performed on DNA from two of the affected siblings uncovered a homozygous missense variant of unknown significance in *NCDN* (c.1297G>C [p.Glu433Gln] [GenBank: NM_014284.3]). Segregation analysis of the variant confirmed that both parents were heterozygous, whereas all three affected siblings, but not a fourth unaffected sibling, were homozygous (Figure 1A). The c.1297G>C variant is predicted to be pathogenic according to MutationTaster and CADD score but tolerated and benign via Sift and PolyPhen-2, respectively (Table S1). Furthermore, c.1297G is conserved with a phastCons score^17^ of 0.884. The expression pattern and known functions of NCDN prompted us to search for additional independent affected individuals with NDD and *NCDN* variants. The identification of disease-causing variants has been improved by development of web-based platforms, enabling researchers to ascertain additional affected individuals with allelic variants and to explore genotype–phenotype correlations. Using GeneMatcher,^18^ a part of the Matchmaker exchange project,^19^ we identified another three simplex affected individuals of French (F2: II.1), Chinese (F3: II.1), and Japanese (F4:II.1) origin carrying *NCDN* variants (Figure 1A). These three independent affected individuals presented with a variable degree of neurodevelopmental delay and carried unique heterozygous *de novo* variants in *NCDN* (c.1433G>A [p.Arg478Glu], c.1492T>C [p.Trp498Arg], and c.1955C>T [p.Pro652Leu] [GenBank: NM_014284.3 for all three variants]), respectively (Figures 1A–1C). The three missense variants were found to have a phastCons score of 1, they localized in highly conserved regions, and they were pathogenic according to the variant intolerance prediction tools MutationTaster, Sift, and PolyPhen-2 (Table S1). Genetic tolerance for each amino acid (aa) position in NCDN (UniProt: Q9UBB6) was further predicted by MetaDome.^20^ The p.Glu433Gln variant is considered “slightly tolerant” (tolerance score = 1.01), while p.Arg478Glu (tolerance score = 0.32), p.Trp498Arg (tolerance score = 0.29), and p.Pro652Leu (tolerance score = 0.4) are considered “intolerant” (Figure 1D). *NCDN* has a missense *Z* score of 3.76 (observed/expected = 0.51 [0.46–0.57]), whereas it has a synonymous *Z* score of 0.73 (o/e = 0.94 [0.83–1.05]), suggesting the gene to be highly intolerant to missense variants. This is supported by a residual variation intolerance score (RVIS) of −1.77, indicating that *NCDN* is among the 4.2% most intolerant human genes to variants.^21^

The clinical features of the three affected individuals with *de novo NCDN* variants overlapped with those of the three affected siblings homozygous for c.1297G>C. The phenotypic findings of the altogether six affected individuals are summarized in Table 1. Neurodevelopmental delay was diagnosed in all affected individuals between ages 1 month and 4 years. Follow-up investigations revealed intellectual disability (ID) that varied from mild to severe. Speech delay was shared by all six affected individuals and developed at age 2–3 years in 5/6 individuals and is still absent in one individual now at 5 years of age. Ability to walk was achieved at age 3–5 years in 3/6 affected individuals. One individual is still unable to walk at age 5 years. Four affected individuals had reached the age of 10 years and showed at that time a capability to read and write corresponding to 2–4 years of delay. Head circumference was reduced (<−2 SD) in 2/6 affected individuals and slightly reduced (<−1 SD) in 4/6 affected individuals. Furthermore, a general growth retardation was found in the three siblings of family 1 (F1:II.1, F1:II.2, and F1:II.3) and in individual F4:II.1. The individual F4:II.1 presented with esotropia, hypertelorism, and epicanthus, but facial dysmorphisms were otherwise absent among the affected individuals. Epilepsy was diagnosed in 5/6 affected individuals and was either generalized (n = 4) or focal (n = 1). The three affected siblings in family 1 had sporadic or febrile seizures, whereas two individuals (F3:II.1 and F4:II.1) had a history of early onset myoclonic encephalopathy. The two affected individuals became seizure free after neurosurgery (F3:II.1) or upon treatment with adrenocorticotropic hormone (ACTH) and Zonisamide (F4:II.1). The electroencephalographic (EEG) patterns showed a slightly pathogenic pattern in the three affected siblings and hypsarrhythmia in affected individuals F3:II.1 and F4:II.1. Magnetic resonance imaging (MRI) performed on three independent affected individuals showed signs of delayed myelination in individual F3:II.1, whereas no overt structural abnormalities were detected in individuals F1:II.1 and F4:II.1.

**Table 1:** Summary of clinical findings in six affected individuals with *NCDN* missense variants

**Family**	**Family 1**			**Family 2**	**Family 3**	**Family 4**
**Individual**	F1:II.1	F1:II.2	F1:II.3	F2:II.1	F3:II.1	F4:II.1
**Gender**	male	female	female	male	male	female
**Age of observed onset**	4 years	4 years	4 years	2.5 years	1 month	4 months
**Age at most recent examination**	16 years	15 years	14 years	13 years	3 years	5 years
**Ethnicity**	Pakistani	Pakistani	Pakistani	French	Chinese	Japanese
***NCDN* variants NM_014284.3**	c.1297G>C (p.Glu433Gln)	c.1297G>C (p.Glu433Gln)	c.1297G>C (p.Glu433Gln)	c.1433G>A (p.Arg478Glu)	c.1492T>C (p.Trp498Arg)	c.1955C>T (p.Pro652Leu)
**Variant type**	homozygous	homozygous	homozygous	*de novo* heterozygous	*de novo* heterozygous	*de novo* heterozygous
**Body height and age**	160 cm at 16 years (−2.5 SD)	149 cm at 15 years (−2.5 SD)	145 cm at 14 years (−2.5 SD)	152 cm at 11 years (+2.0 SD)	96 cm at 3 years (+0.7 SD)	92 cm at 5 years (−3.2 SD)
**Head circumference and age at measurement**	53 cm at 14 years (−1.1 SD)	50 cm at 13 years (−2.8 SD)	50 cm at 12 years (−2.6 SD)	51 cm at 10 years (−1.5 SD)	47 cm at 3 years (−1.6 SD)	49 cm at 5 years (−0.8 SD)
**Facial dysmorphisms**	no	no	no	no	high arched palate	esotropia, hypertelorism, epicanthus
**Ambulation**	walked at age 3 years, normal ambulation	walked at age 5 years, normal ambulation	walked at age 1 year, normal ambulation	walked at age 14 months, normal ambulation	walked at age 3 years, normal ambulation	unable to walk at age 5 years
**Speech development**	first words at age 2–3 years, normal speech at age 16 years	first words at age 2 years, normal speech at age 15 years	first words at age 2 years, normal speech at age 14 years	first words at age 3 years, profound speech problems at age 13 years	first words at age 2 years	no meaningful words at age 5 years
**Learning disabilities**	read and write with difficulties at age 16 years	read and write with difficulties at age 15 years	read and write with difficulties at age 14 years	read and write like a beginner at age 10 years	N/A	N/A
**Vision, hearing**	normal	normal	normal	normal	normal	normal
**ID**	yes, mild	yes, mild	yes, mild	yes, mild	yes, moderate	yes, severe
**Seizures**	yes	yes (febrile)	yes (febrile)	no	yes	yes
**Frequency**	1–2/month	rare	rare	–	50 times/day prior to treatment	5 times/day prior to treatment
**Type of seizures**	generalized	generalized, fever induced	generalized, fever induced	–	epileptic spasms, focal	epileptic spasms, myoclonus
**Medication**	valproic acid	no medication	valproic acid	–	valproic acid, topiramate, perampanel, and ACTH; seizure-free after surgery	seizure-free with ACTH and ZNS
**Brain MRI**	normal structures	not performed	not performed	not performed	delayed myelination	normal structures
**EEG**	slightly abnormal	slightly abnormal	slightly abnormal	normal at 3 years	hypsarrhythmia at age 4 months	hypsarrhythmia at age 1 year

N/A, not available; ACTH, adrenocorticotropic hormone; ZNS, zonisamide.

**Figure 2:**
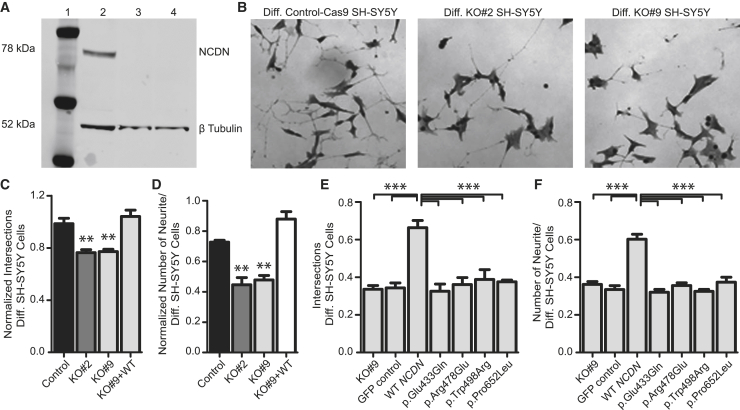
Missense variants in *NCDN* alter length and number of neurites in SH-SY5Y cells (A) Immunoblot analysis of WT SH-SY5Y (lane 2) and the two independent SH-SY5Y cell clones, KO#2 (lane 3) and KO#9 (lane 4), with CRISPR/Cas9-induced homozygous deletions in *NCDN*, confirming depletion of NCDN protein. An *Anti-Ncdn* antibody (Sigma-Aldrich) was used with β-tubulin (Sigma-Aldrich) as a loading control. A PageRuler Plus pre-stained protein ladder (lane 1) was used as molecular weight markers (Thermo). (B) Bright field microscopy images of control-Cas9 SH-SY5Y cells (left) and the two independent SH-SY5Y clones, KO#2 and KO#9 (mid and right). Cells were differentiated (Diff.) for one week. (C) Neurite length in differentiated SH-SY5Y lines. The clones KO#2 and KO#9 show reduced neurite length when compared to the control clone (Cas9-transfected without gRNA). Neurite length was completely rescued in KO#9 after expression of WT *NCDN.* Data were normalized to WT SH-SY5Y cells differentiated in parallel and set to 1.0. (D) Neurite number in differentiated SH-SY5Y lines. Cells of clones KO#2 and KO#9 showed a reduced neurite number when compared to the Cas9-transfected control clone. Complete restoration of neurite numbers was observed in KO#9 cells after expression of WT *NCDN.* Data were normalized as in (C). (E) Overexpression of WT *NCDN* and *NCDN* containing the four missense variants in KO#9 SH-SY5Y line. Expression of WT NCDN rescued the neurite length in KO#9. In contrast, overexpression of *NCDN* encoding the missense variants p.Glu433Gln, p.Arg478Glu, p.Trp498Arg, and p.Pro652Leu failed to rescue the reduced neurite length in KO#9. The transfection control (GFP control) showed a neurite outgrowth similar to KO#9. (F) Expression of WT NCDN rescued the neurite number in KO#9. Overexpression of the four *NCDN* variants in KO#9 failed to rescue the reduced number of neurites. The transfection control (GFP control) showed a neurite number similar to KO#9. The experiments were performed in triplicate and data are shown as mean ± standard error of the mean represented by error bars. Statistical analysis of three independent experiments was performed by one-way ANOVA with Dunnett’s multiple-comparisons test (^∗∗^p < 0.01; ^∗∗∗^p < 0.001).

To clarify the pathophysiological effects of the observed *NCDN* variants, we then generated a neural cell model depleted of NCDN by using the human neuroblastoma line SH-SY5Y. When stimulated with retinoic acid (RA), wild-type (WT) SH-SY5Y cells acquire neuron-like phenotypes such as neurite formation, electrical excitability, and expression of neurotransmitters and neurotransmitter receptors.^22^ We targeted *NCDN* in WT SH-SY5Y cells by using CRISPR/Cas9 and expanded two independent clones with distinct homozygous *NCDN* deletions, one spanning 16 bp (c.1458_1473del16 [GenBank: NM_014284.3]; SH-SY5Y^*NCDN*Δ16/Δ16^), assigned KO#2, and one spanning 1,158 bp; (chr1: 36,027,723–36,028,880; SH-SY5Y^*NCDN*Δ1158/Δ1158^), assigned KO#9. The 16 bp deletion (KO#2) is located within exon 5 of *NCDN* and the 1,158 bp deletion (KO#9) spans intron 3 to exon 5 (Figure S1). Both deletions are predicted to cause frameshift and the premature stop codons p.Ala486Alafs^∗^45 and p.Val382Alafs^∗^24, respectively. We also generated a control, assigned “control-Cas9,” by transfecting WT SH-SY5Y cells with CRISPR/Cas9 but without gRNA. The top predicted off-target sites were analyzed by Sanger sequencing and revealed WT sequences in the KO#2 and KO#9 clones (Figure S2). Furthermore, depletion of NCDN protein was confirmed in both clones by immunoblot analysis (Figure 2A). Given the effects of NCDN on neurogenesis,^16^ we then differentiated the control-Cas9, KO#2, and KO#9 lines for 7 days with RA, allowing for neurite formation (Figure S3).^22^ Image-based analysis revealed that neurites were formed in all three lines (Figure 2B). We then estimated the average neurite length in each line by counting intersections between neurites and test lines of a superimposed frame of fixed size^23^ applied on neuronal cultures as described previously.^24^ The number of neurites intersecting with test lines were divided by the total number of soma present in the image and used as a measure of neurite outgrowth. The average number of neurites per cell was manually counted in individual cells from a subset of all collected images via the cell counter plugin of ImageJ software (Fiji v.1.52p). We found that the neurites in the two NCDN-deficient lines were shorter and fewer in number when compared with neurites in the control-Cas9 line expressing endogenous NCDN. To clarify whether the altered neurite outgrowth was a direct effect of *NCDN* deficiency, we then expressed WT NCDN in the KO#9 clone. Transfection with a full-length WT *NCDN* construct (pAcGFP1-N1-*NCDN*-WT) followed by differentiation revealed a restoration of both length and numbers of neurites in KO#9 (Figures 2C and 2D). We next investigated whether the four *NCDN* missense variants of our affected individuals could restore the neurite length and number in SH-SY5Y cells depleted of *NCDN*. To this end, KO#9 cells were transfected with full-length *NCDN* expression constructs containing any of the four *NCDN* variants: c.1297G>C (p.Glu433Gln), c.1433G>A (p.Arg478Glu), c.1492T>C (p.Trp498Arg), or c.1955C>T (p.Pro652Leu). Transfection with a WT *NCDN* construct was used as reference. At 24 h post-transfection, cells were differentiated for 7 days via RA stimulation. Image-based analysis of KO#9 cells transfected with each of the four variant constructs revealed that both length and number of neurites were consistently reduced when compared with KO#9 cells transfected with WT *NCDN* (Figures 2E and 2F). The four variants thus failed to rescue the neurite phenotypes that were comparable to non-transfected KO#9 cells. Furthermore, neurite formation in KO#9 cells transfected with pAcGFP1-N1-empty vector were similar to non-transfected KO#9 cells (Figures 2E and 2F). These data suggest that the impaired neurite formation in SH-SY5Y cells depleted of NCDN is rescued by the expression of WT NCDN but not by *NCDN* constructs expressing the four missense variants.

**Figure 3:**
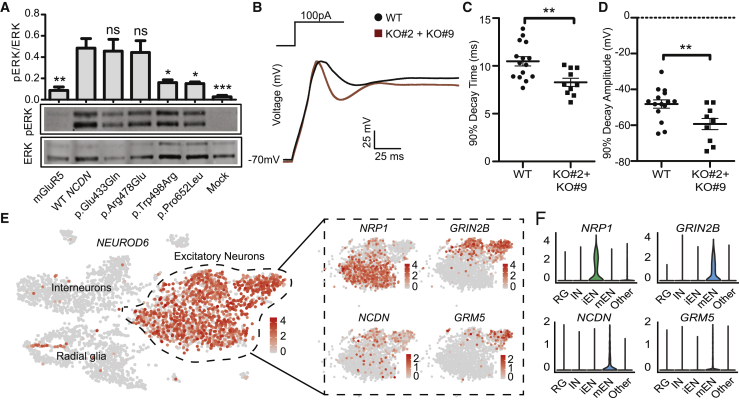
*NCDN* variants interfere with mGluR5 signaling and alter electrophysiological properties of SH-SY5Y cells (A) ERK1/2 phosphorylation in transfected KO#9 cells expressing WT NCDN or NCDN containing each of the p.Glu433Gln, p.Arg478Glu, p.Trp498Arg, and p.Pro652Leu variants when compared to cells expressing only mGluR5. DHPG-induced extracellular signal-regulated kinase (ERK) phosphorylation was quantified in serum-starved transfected KO#9 cells expressing the mGluR5 receptor alone, mGluR5 receptor co-expressed with WT NCDN, or co-expressed with NCDN containing p.Glu433Gln, p.Arg478Glu, p.Trp498Arg, and p.Pro652Leu. The p.Glu433Gln and p.Arg478Glu rescued phosphorylation to normal levels, whereas p.Trp498Arg and p.Pro652Leu, positioned in the mGluR5 interacting domain, did not. The phosphorylation of ERK1/2 was determined by immunoblot and band intensities correspond to phospho-ERK1/2 and total ERK1/2. Phosphorylated ERK1/2 was normalized with total ERK1/2. Data are shown as mean ± standard error of the mean represented by error bars. Statistical analysis of four independent experiments was performed by one-way ANOVA with Dunnett’s multiple-comparisons test (^∗^p < 0.05; ^∗∗^p < 0.01; ^∗∗∗^p < 0.001; ns = not significant). Mock = un-transfected. Representative immunoblot (n = 5, bottom panels) of DHPG-induced ERK phosphorylation. (B) Abortive action potentials (APs) evoked in response to rectangular current injections in differentiated SH-SY5Y cells with a holding potential of −70 mV and current injections of 100 pA for 100 ms, suggesting different decay kinetics between NCDN-depleted (KO#2 [n = 5], KO#9 [n = 5]) and WT (n = 15) SH-SY5Y cells. The average rising time was similar when comparing KO#2 and KO#9 (32.18 ± 0.74 ms) with WT SH-SY5Y (33.35 ± 0.64 ms) cells at injected currents of 100 pA. The average voltage responses show abortive AP. (C and D) 90% AP decay time (C) and 90% AP decay amplitude (D) show a significant change in AP decay kinetics in KO#2 and KO#9 SH-SY5Y cells when compared to WT SH-SY5Y cells. Data are shown as mean ± standard error of the mean represented by error bars. Statistical analysis was carried out with unpaired t tests with Welch correction (^∗∗^p < 0.005). mV, millivolts; ms, milliseconds. (E) Cell-type-specific expression of *NCDN* in the human cortex derived from fetuses at post-conceptional weeks 6–37. The t-SNE plot shows the excitatory neural cell cluster defined by *NEUROD6* expression (left panel). The cluster is further divided into immature excitatory neurons (iENs), expressing *NRP1,* and maturing excitatory neurons (mENs), expressing *GRIN2B* (boxed). Expression of *NCDN* and *GRM5* are detected mainly at the mEN stage. Expression levels are scaled, log-normalized data from Seurat 3. Expression levels are color-coded in the feature plots. (F) Expression levels of the five genes in the excitatory cell cluster of fetal cortex showing enriched expression of *NCDN* and *GRM5* in mEN. RG, radial glia; IN, interneurons; iEN, immature excitatory neurons; mEN, maturing excitatory neurons.

NCDN interacts with mGluR1 and mGluR5, encoded by *GRM1* (MIM: 604473) and *GRM5* (MIM: 604102).^14^^,^^25^ Activation of mGluR5 induces the phosphorylation of extracellular signal-regulated kinase (ERK)^26^ that can be used as a measure of mGluR activity.^27^ We therefore reasoned that the effect of *NCDN* variants on mGluR1/5-induced signaling can be assessed by quantification of ERK phosphorylation. To this end, GFP-tagged WT *NCDN* and each of the four mutant constructs were co-transfected with a full-length *GRM5* construct into KO#9 cells. The *GRM5* construct transfected into KO#9 cells was used as control. Transfection efficiency was confirmed by fluorescence after 24 h, followed by serum starvation for 24 h. Activation of mGluRs via dihydroxyphenylglycine (DHPG) was induced, followed by cell harvest. Immunoblot analysis of protein lysates from the cells revealed a significant increase in ERK1/2 phosphorylation in cells expressing WT *NCDN* when compared with cells expressing only *GRM5* (Figure 3A). Compared with WT *NCDN*, the overexpression of *NCDN* constructs containing the c.1492T>C or c.1955C>T variants showed markedly reduced levels of phosphorylated ERK1/2 when assessed by immunoblotting. This observation is consistent with the position of the two missense variants in a region of *NCDN* encoding the mGluR5-interacting domain (Figures 1D and 3A).^14^^,^^28^ In contrast, overexpression of the *NCDN* variants c.1297G>C and c.1433G>A, replacing residues outside the mGluR5-interacting domain of NCDN, showed no apparent effect on ERK1/2 phosphorylation (Figure 1A). Besides the interaction with mGluR5, NCDN tethers or mediates the activation of several proteins important for downstream signaling through phosphorylation of both ERK and CaMKII.^14^^,^^15^^,^^29^ We therefore sought to investigate the effect of loss of NCDN in SH-SY5Y on the interacting partners mGluR1 and mGluR5, as well as on calmodulin-dependent kinase II (CaMKII). Using qPCR, we observed differential expression for the three NCDN-interacting partners when comparing *NCDN*-deficient and WT SH-SY5Y cells (Figure S4). Our findings suggest that the two variants c.1492T>C and c.1955C>T, affecting residues in the mGluR-interacting domain, inhibit mGluR1/5-induced ERK phosphorylation and that NCDN depletion in SH-SY5Y cells results in altered expression of factors belonging to the mGluR pathway.

Seizure was a shared feature in 5/6 affected individuals in our study. Furthermore, mice with a homozygous conditional disruption of *Ncdn* in the central nervous system exhibit epilepsy.^15^ We therefore set out to investigate the electrophysiological properties of *NCDN*-deficient SH-SY5Y cells by using whole-cell patch-clamp recordings. Cultures of KO#9 and WT SH-SY5Y lines were differentiated for 7 days. Cell sizes from both cultures were similar when evaluated by capacitance measurements (Figure S5A). The patch-clamp analysis revealed a resting membrane hyperpolarization in KO#2 and KO#9 lines (−45.00 ± 0.55 mV) when compared to WT SH-SY5Y (−38.23 mV ± 1.58 mV; Figure S5B). Upon 100 ms rectangular current injections, NCDN-depleted lines showed abortive action potentials (APs) and without differences in the rising phase of the APs (Figure 3B). However, AP repolarization kinetics was significantly different, and there was an increased repolarization rate (mV/ms) in *NCDN*-deficient cells in response to different injected currents (Figures 3C, 3D, and S6). These observations strongly suggest altered electrophysiological properties in *NCDN*-deficient and differentiated SH-SY5Y cells, consistent with seizures in mice with conditional depletion of *Ncdn*.

We then sought to investigate the expression pattern of *NCDN* in human fetal cortex by using available and previously published data.^30^ In this data, we observed that *NCDN* expression is enriched in excitatory neural cells defined by *NEUROD6* (MIM: 611513) expression (Figure 3E). Specifically, the expression of *NCDN* is predominantly found in maturing excitatory neurons (mENs), defined by expression of the glutamate ionotropic receptor gene *GRIN2B* (MIM:138252), but not in immature excitatory neurons (iENs), defined by *NRP1* (MIM: 602069) expression (Figure 3F).^30^ Similarly, analysis of *GRM5* expression showed expression levels confined to the mEN stage (Figures 3E and 3F). These data show that *NCDN* is highly expressed in mENs of the fetal cortex together with *GRM5,* consistent with interactions between NCDN and mGluR5.

Our series of six affected individuals with *NCDN* variants shared several core NDD features such as learning disability and ID, delayed speech development, and variable degrees of reduced head circumference. While all four *NCDN* variants failed to rescue the impaired neurite formation in NCDN-depleted SH-SY5Y cells, the inheritance, onset, severity, and combination of symptoms varied between families without shared distinctive clinical features among affected individuals. The three siblings (F1:II.1, F1:II.2, and F1:II.3) with a bi-allelic *NCDN* variant c.1297G>C presented with a uniform phenotype with growth retardation, mild ID, and sporadic or febrile seizures. Their heterozygous parents had neither neurological symptoms nor cognitive impairment. Individual F2:II.1, carrying a heterozygous *de novo* variant c.1433G>A, showed a similar mild ID and speech delay but no seizures. On the other hand, individuals F3:II.1 and F4:II.1 with the *de novo* variants c.1492T>C and c.1955C>T, respectively, showed a more severe form of developmental delay with an earlier onset, moderate to severe ID, and epileptic spasms in our series of patients. Interestingly, *in vitro* expression of the c.1492T>C and c.1955C>T variants, situated in the mGluR5-interacting domain,^14^^,^^28^ interfered with mGluR5-induced ERK phosphorylation in our assay. On the basis of a combination of our clinical observations and experimental data, it is thus tempting to speculate that the c.1297G>C and c.1433G>A variants, without ability to promote neurite formation and with no detectable effects on ERK phosphorylation in our assays, alter NCDN functions in slightly different ways than the c.1492T>C and c.1955C>T variants, showing a reduced mGluR5-mediated ERK phosphorylation (Figure 3A). In a prior report, three individuals were identified with heterozygous *de novo* deletions spanning 1.1 Mb to 3.1 Mb involving *NCDN*.^31^ The three affected individuals presented with mild to moderate ID and motor and speech delay. However, seizures were not reported, suggesting that *NCDN* haploinsufficiency does not necessarily cause epilepsy. It may therefore be hypothesized that the seizures, associated with three missense variants in our study, define a sub-phenotype mediated by a dominant-negative effect. The variant *NCDN* allele c.1297G>C in family 1 should in such a case be hypomorphic to explain the silent phenotype in heterozygous parents. The nucleotide position c.1297G has a lower phastCons score (0.884) and the corresponding aa residue is more tolerated (MetaDome tolerance score) when compared with the aa residues corresponding to the three positions c.1433G, c.1492T, and c.1955C, consistent with hypomorphism as a plausible explanation. Domain-specific missense variants leading to alternate mechanisms and phenotypic pleiotropy have recently been reported. Specific bi-allelic variants in the transcription factor gene *DEAF1* (MIM: 602635) appear to cause hypomorphic loss-of-function alleles, whereas some heterozygous *de novo* variants have a dominant-negative (antimorph) effect.^32^ In *TRIO* (MIM: 609701), encoding a guanine nucleotide exchange factor, domain-specific missense variants mediate either haploinsufficiency or gain of function leading to distinct phenotypes.^33^ Similar to DEAF1 and TRIO, NCDN interacts with multiple proteins and impacts different functions of the central nervous system.^13^^,^^34^^,^^35^ We therefore suggest that the four different missense variants may cause distinct and domain-specific changes of NCDN functions, ultimately leading to the phenotypic variability. A detailed understanding on how the identified *NCDN* variants interfere with binding to different protein partners will now require further investigations.

In conclusion, we identified four different missense variants in *NCDN* that are functionally relevant in six individuals presenting with a variable degree of neurodevelopmental delay, ID, and seizures. Five out of the six affected individuals were diagnosed with variable types of epilepsy, a core phenotypic feature of NDD with a 10-fold increased prevalence in individuals with ID compared with the general population.^36^ Three *NCDN* variants occurred *de novo*, whereas one variant segregated in a bi-allelic state in three siblings with a uniform clinical presentation. Functional characterization of the four *NCDN* variants showed that they, in contrast to WT *NCDN*, were unable to rescue the impaired neurite formation in NCDN-depleted SH-SY5Y cells. Furthermore, mGluR signaling, measured by ERK phosphorylation levels, was compromised by *NCDN* variants located in the mGluR5-binding domain of the protein in two individuals presenting with severe clinical features of NDD. Despite the functional effects shown for the four *NCDN* variants in our assays, the precise mechanisms leading to the variable clinical presentations and different inheritance patterns remain elusive. Mice with conditional loss of *Ncdn* in the central nervous system exhibit learning defects and epilepsy. These features are concordant with the phenotype in five of our affected individuals, however they are not mediated by complete loss of function. Furthermore, large heterozygous deletions in patients with NDD suggest NCDN haploinsufficiency is not associated with epilepsy. However, a dominant-negative effect, in particular of the c.1297G>C and c.1433G>A variants, is plausible and would require the recessive variant c.1297G>C to be hypomorphic. Given that NCDN interacts with multiple partners in the central nervous system during development and that the *NCDN* variants identified may affect these interactions differently, we therefore hypothesize that the variants interfere with NCDN functions in different ways, ultimately leading to variable clinical features. However, the precise mechanistic explanations for the variable molecular, cellular, and clinical effects of the monoallelic and bi-allelic *NCDN* variants warrant further investigations, requiring mapping of functional domains of NCDN and its 3D structure.

## Declaration of interests

The authors declare no competing interests.

## Data and code availability

The *NCDN* variants with accession numbers are available at https://www.ncbi.nlm.nih.gov/clinvar/ (GenBank: NM_014284.3) for variants c.1297G>C: SCV001443681, c.1433G>A: SCV001443682, c.1492T>C: SCV001443683, and c.1955C>T: SCV001443684. WES datasets have not been deposited in a public repository because of privacy and ethical restrictions but are available from the corresponding author on request.

## Web resources

CRISPR direct, https://crispr.dbcls.jp/
GeneMatcher, https://genematcher.org/
Genic Intolerance Database, http://genic-intolerance.org/
GnomAD, https://gnomad.broadinstitute.org/
MetaDome, https://stuart.radboudumc.nl/metadome
MutationTaster, http://www.mutationtaster.org/
OMIM, https://omim.org/
Primer 3 (v.0.4.0), https://bioinfo.ut.ee/primer3-0.4.0/
The National Center for Biotechnology Information (NCBI), https://www.ncbi.nlm.nih.gov/
UCSC Cell Browser (human cerebral cortex), https://cells.ucsc.edu/
Variant Effect Predictor (VEP) from ENSEMBL, https://www.ensembl.org/

## Supplemental information

Document S1. Supplemental note, Figures S1–S6, Tables S1–S3, and supplemental methods

Document S2. Article plus supplemental information
