# Monoallelic and bi-allelic variants in *NCDN* cause neurodevelopmental delay, intellectual disability, and epilepsy

**DOI:** 10.1016/j.ajhg.2022.02.007

**Published:** 2022-03-03

**Authors:** Ambrin Fatima, Jan Hoeber, Jens Schuster, Eriko Koshimizu, Carolina Maya-Gonzalez, Boris Keren, Cyril Mignot, Talia Akram, Zafar Ali, Satoko Miyatake, Junpei Tanigawa, Takayoshi Koike, Mitsuhiro Kato, Yoshiko Murakami, Uzma Abdullah, Muhammad Akhtar Ali, Rein Fadoul, Loora Laan, Casimiro Castillejo-López, Maarika Liik, Zhe Jin, Bryndis Birnir, Naomichi Matsumoto, Shahid M. Baig, Joakim Klar, Niklas Dahl

## Main text

(The American Journal of Human Genetics *108*, 739–748; April 1, 2021)

In the originally published version of this article, family 2 was assigned an incorrect aa substitution as a result of the G>A transition in codon 478 of the *NCDN* gene. The aa substitution in individual F2: II.1 should read p.Arg478Gln, not p.Arg478Glu. The error appeared consistently throughout the text, in Table 1, and in Figures 1, 2, and 3; the error has been corrected online and Figures 1, 2, and 3 appear correctly here as well. The authors regret this error.

**Figure:**
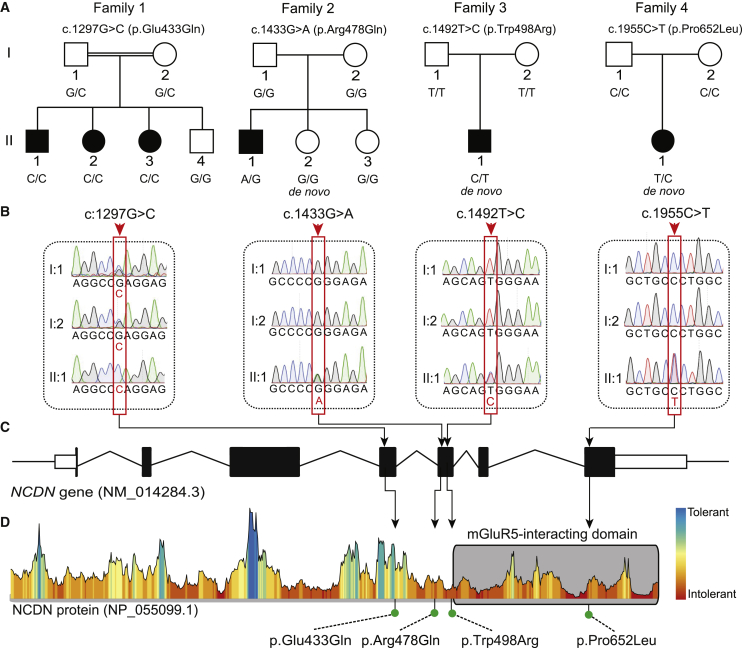
Figure 1. Segregation of rare *NCDN* missense variants in families with neurodevelopmental phenotypes (corrected)

**Figure:**
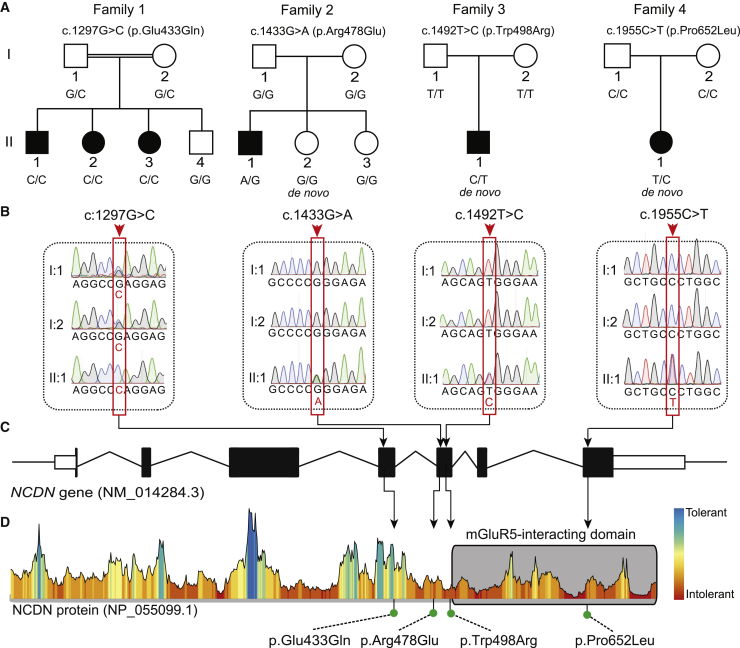
Figure 1. Segregation of rare *NCDN* missense variants in families with neurodevelopmental phenotypes (original)

**Figure:**
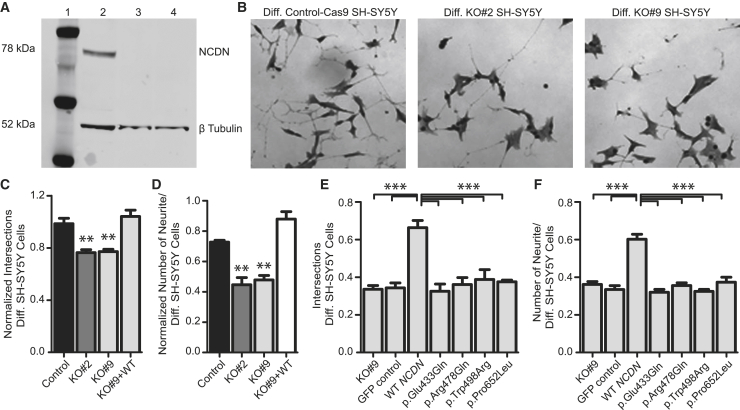
Figure 2. Missense variants in *NCDN* alter length and number of neurites in SH-SY5Y cells (corrected)

**Figure:**
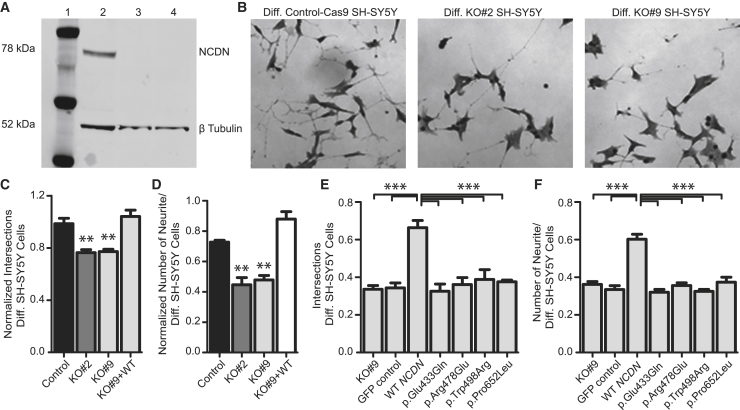
Figure 2. Missense variants in *NCDN* alter length and number of neurites in SH-SY5Y cells (original)

**Figure:**
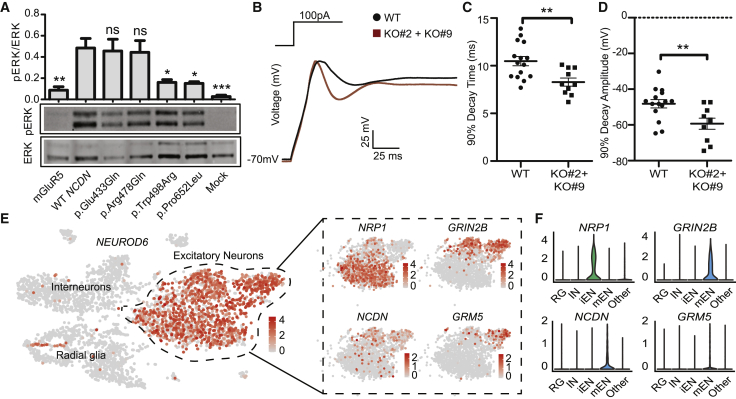
Figure 3. *NCDN* variants interfere with mGluR5 signaling and alter electrophysiological properties of SH-SY5Y cells (corrected)

**Figure:**
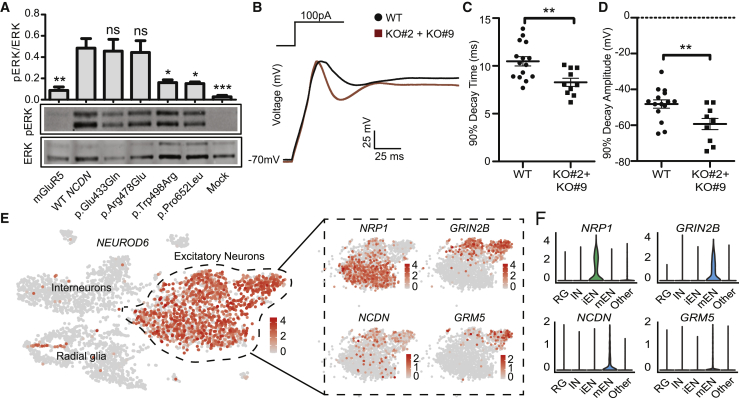
Figure 3. *NCDN* variants interfere with mGluR5 signaling and alter electrophysiological properties of SH-SY5Y cells (original)

