# Supplementary material for: Monoallelic and bi-allelic variants in NCDN cause neurodevelopmental delay, intellectual disability, and epilepsy
Source: Am J Hum Genet. 2021 Mar 11;108(4):739–48. doi: 10.1016/j.ajhg.2021.02.015 (PMC8059333; doi:10.1016/j.ajhg.2021.02.015)
Supplement: Document S1. Supplemental note, Figures S1–S6, Tables S1–S3, and supplemental methods [file mmc1.pdf]

The American Journal of Human Genetics, Volume *108*

Supplemental information

Monoallelic and bi-allelic variants in *NCDN* cause neurodevelopmental delay, intellectual disability, and epilepsy

Ambrin Fatima, Jan Hoeber, Jens Schuster, Eriko Koshimizu, Carolina Maya-Gonzalez, Boris Keren, Cyril Mignot, Talia Akram, Zafar Ali, Satoko Miyatake, Junpei Tanigawa, Takayoshi Koike, Mitsuhiro Kato, Yoshiko Murakami, Uzma Abdullah, Muhammad Akhtar Ali, Rein Fadoul, Loora Laan, Casimiro Castillejo-López, Maarika Liik, Zhe Jin, Bryndis Birnir, Naomichi Matsumoto, Shahid M. Baig, Joakim Klar, and Niklas Dahl

Supplemental case report

Family 1 (F1:II.1, F1:II.2 and F1:II.3) was recruited from National Institute for Biotechnology and Genetic Engineering, Faisalabad, Pakistan (S.M.). Additional cases were enrolled from Pitié-Salpêtrière Hospital, Paris, France (C.M. and B.K., case F2:II.1), and Yokohama City University Graduate School of Medicine, Japan (E.K. and N.M., cases F3:II.1 and F4:II.1). Venous blood samples were obtained from participants and genomic DNA was extracted according to standard protocols. All participating research centers were connected through the public data sharing platform GeneMatcher (entry "NCDN").

Supplemental figures

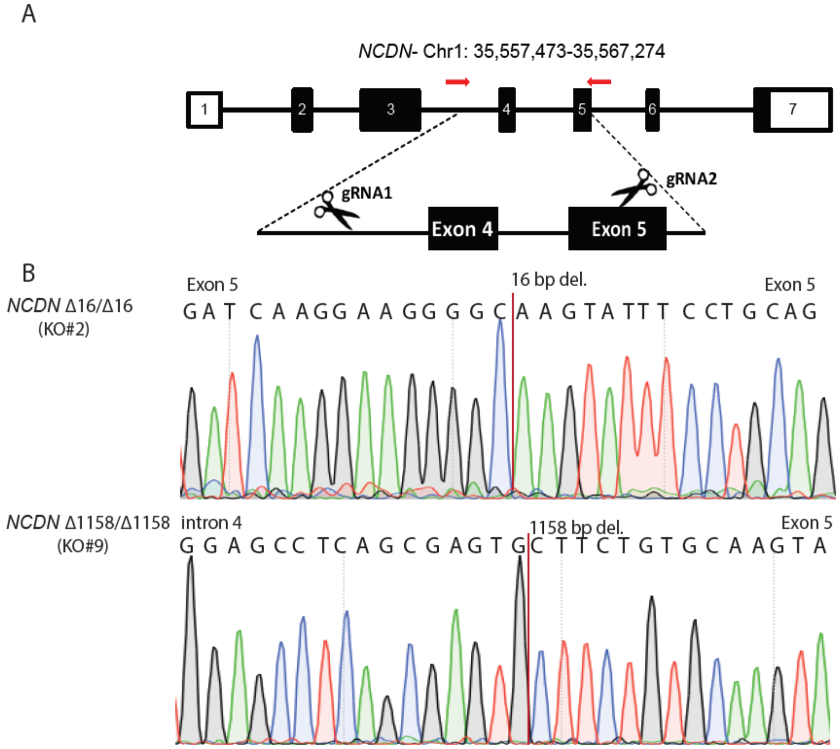

Figure S1. **Schematic presentation of CRISPR/Cas9 targeting of *NCDN* in SH-SY5Y cells.** (A) Relative positions of *NCDN* guide RNAs (gRNAs) designed to target intron 3 and exon 5 of the *NCDN* gene. Screening of SH-SY5Y cell clones edited for *NCDN* was carried out using PCR primers flanking both gRNAs (red arrows) and in-between both gRNAs (not shown) in different combinations. (B) Sanger sequencing of *NCDN* clones derived from single cells revealed a homozygous deletion of 16 bp in exon 5 (clone *NCDN*Δ16/Δ16, assigned KO#2), and a 1158 bp deletion spanning intron 3 to exon 5 (clone *NCDN*Δ1158/Δ1158, assigned KO#9).

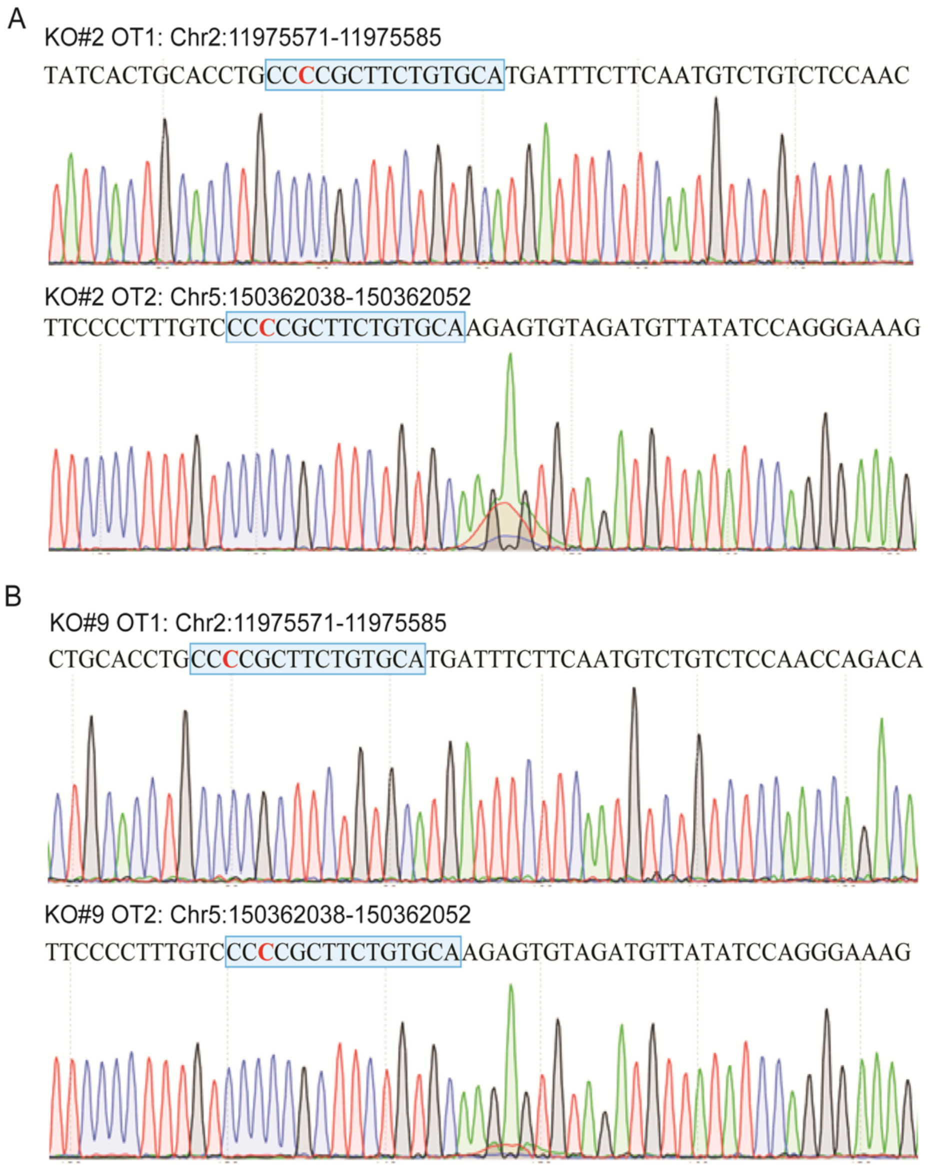

Figure S2. Analysis of potential off-target sites by Sanger sequencing in SH-SY5Y clones KO#2 and KO#9.

Sequence chromatogram of the two top-predicted off targets (*OT1* and *OT2*) in (A) KO#2 cells and (B) KO#9 cells. Matched sequences are highlighted in blue and mismatch nucleotides are marked in red.

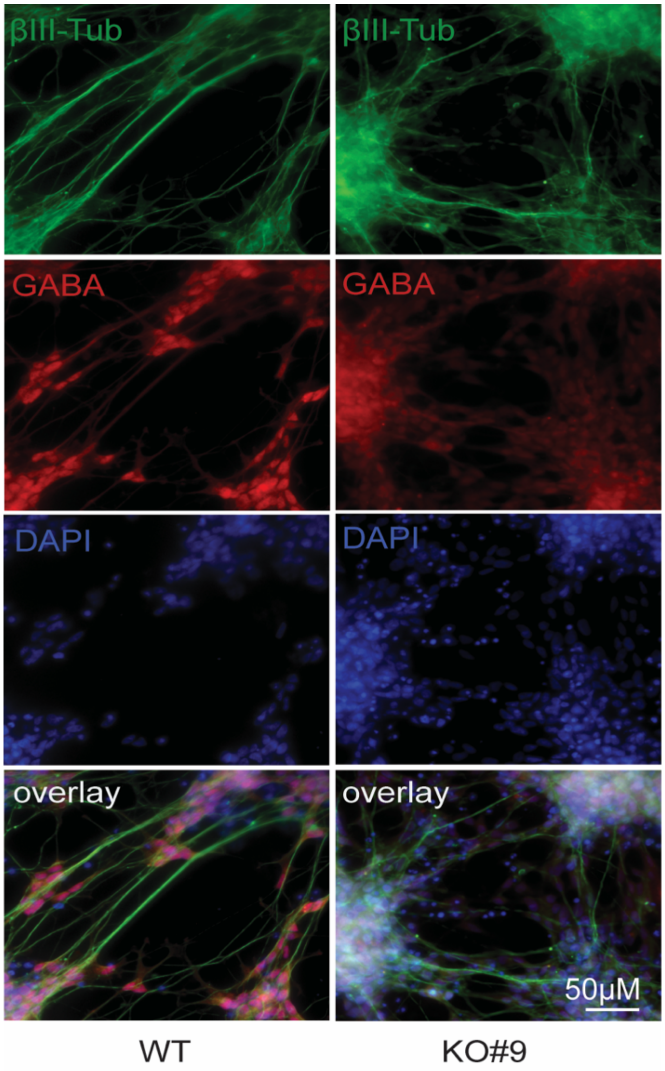

Figure S3. Differentiation and staining of SH-SY5Y cells.
Representative images of WT and KO#9 SH-SY5Y cells differentiated for seven days and stained for βIII-Tubulin (green) and GABA (red). Cells are counterstained with the nuclear marker DAPI (blue). Stainings are merged at the bottom. Size bar: 50 µm.

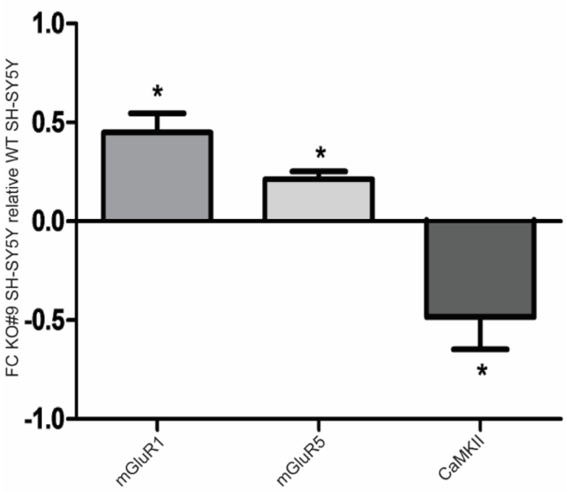

Figure S4. Depletion of NCDN in SH-SY5Y cells alters expression of genes encoding proteins in the group I mGluR pathway.

Expression of genes encoding the NCDN interacting partners mGluR1, mGluR5 and Calmodulin Dependent Kinase II (CaMKII), analyzed by RT-qPCR in un-differentiated KO#9 cells. Expression levels are presented as fold change (FC) values corresponding to $\log_2(2^{-\Delta\Delta C_T})$ after normalization to GAPDH in WT SH-SY5Y. A FC of zero indicates similar expression in WT SH-SY5Y and KO#9. Data was obtained from three biological replicates. Error bars correspond to SEM. Statistical analysis was carried out with unpaired Students t-tests with Welch's correction (*$p < 0.05$).

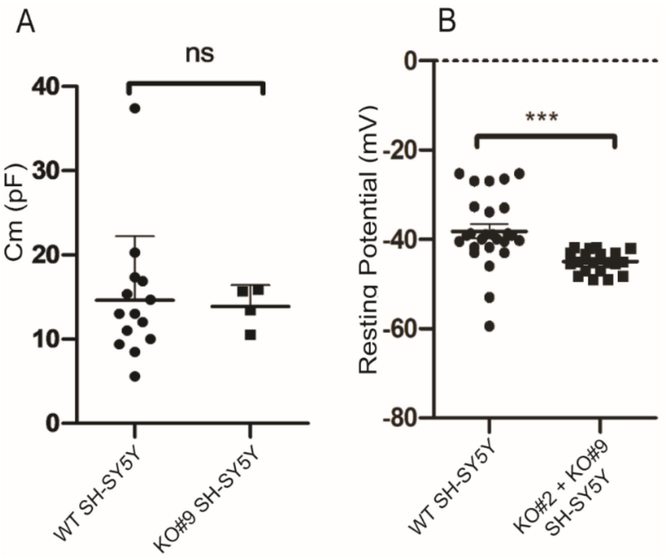

Figure S5. Passive membrane properties of WT SH-SY5Y and NCDN deficient SH-SY5Y cells from patch-clamp measurements.

(A) Membrane capacitance, as a measurement of cell surface area, of WT SH-SY5Y (n=14) and KO#9 SH-SY5Y cells (n=4). Cells were differentiated for one week with RA before being measured. The results indicate similar sizes for WT and NCDN deficient SH-SH5Y cells. (B) Resting membrane potential in cells differentiated for one week with RA. Significant resting membrane hyperpolarization is observed in the NCDN deficient SH-SY5Y cells KO#2 and KO#9 (n=20) when compared to WT SH-SH5Y (n=26). Error bars represent SEM; Cm: cell capacitance; pF: picofarads; mV: millivolts. Statistical analysis was carried out using unpaired t-tests with Welch correction (***$p<0.0005$).

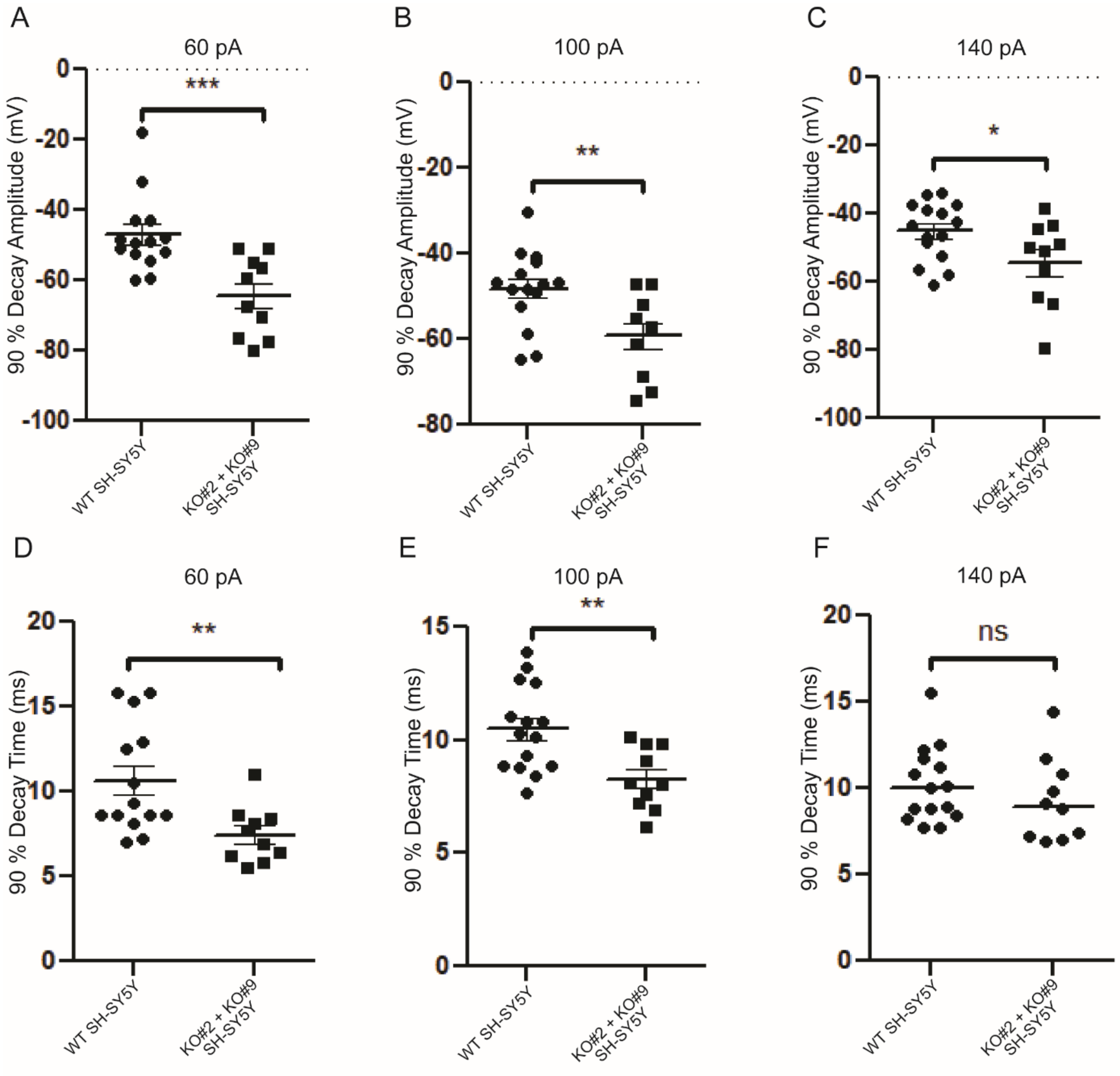

Figure S6. **Kinetics of action potential repolarization.**
Whole-cell patch clamp recordings of WT SH-SY5Y (n=14) and NCDN deficient SH-SY5Y cells (KO#2: n=5 and KO#9: n=5) after stimulus-dependent impulses. Cells were stimulated with rectangular currents of (A) 60 pA (B) 100 pA and (C) 140 pA for 100 ms at a holding potential of -70 mV. Action potential decay amplitude was reduced in NCDN deficient SH-SY5Y cells using all three conditions. (D-F) Action potential decay time was found reduced in NCDN deficient SH-SY5Y cells at (D) 60 pA (E) 100 pA and (F) 140 pA. The decay amplitude corresponds to the voltage change of the rising phase of the AP and the decay time defines the time between the AP-peak and end of repolarization state. Error bars represent SEM. Statistical analyses were carried out by unpaired t-tests with Welch correction (*p<0.05; **p<0.005; ***p<0.0005).

Supplemental tables

Table S1. Pathogenicity prediction of *NCDN* (NM_014284.3) variants by different *in silico* tools.

Variation (hg19)	Mutation Taster	Sift	Sift Score	PolyPhen-2	HumVar	PhyloP	PhastCons	CADD
1:36028146;c.1297G>C	disease causing	tolerated	0.57	benign	0.216	2.158	0.884	21.5
1:36028850;c.1433G>A	disease causing	damaging	0	probably damaging	0.997	5.029	1	28.8
1:36028909:c.1492T>C	disease causing	damaging	0	probably damaging	0.997	1.569	1	26.0
1:36031029:c.1955C>T	disease causing	damaging	0.05	possibly damaging	0.766	3.530	1	24.5

Table S2. Primers used in this study.

Methods	Primer name	5' – 3' sequence
CRISPR-Cas9	*NCDN-grna1-Top*	CACCGCTGGAGCCTCAGCGAGTACG
	NCDN-grna1-Bottom	AAACCGTACTCGCTGAGGCTCCAGC
	NCDN-grna2-Top	CACCgCCTCGCTTCTGTGCAAGTAT
	NCDN-grna2-Bottom	AAACATACTTGCACAGAAGCGAGGc
Site directed mutagen.	*NCDN-p.E433Q-F*	CTACGAGGAGGCCCAGGAGGCCAATGA
	NCDN-p.E433Q-R	TCATTGGCCTCCTGGGCCTCCTCGTAG
	NCDN-p.R478E-F	GAAGATGGGCCCCAGGAGATCCTGATCA
	NCDN-p.R478E-R	TGATCAGGATCTCCTGGGGCCCATCTTC
	NCDN-p.W498R-F	TATTTCCTGCAGCAGCGGGAACTCACATCCC
	NCDN-p.W498R-R	GGGATGTGAGTTCCCGCTGCTGCAGGAAATA
	NCDN- p.P652L -F	GTGTGCCTCTGCTGCTCTGGCTGGCCCCCG
	NCDN- p.P652L -R	CGGGGGCCAGCCAGAGCAGCAGAGGCACAC
	NCDN- p.P652L -R	CGGGGGCCAGCCAGAGCAGCAGAGGCACAC
qPCR	*CAMKIIB-F*	TGTCAGCCAGAGATCACCAG
	CAMKIIB-R	ACTCTCTCGCCACAATGTCT
	CaM2-F	GGCACAATTGACTTCCCTGA
	CaM2-R	GCGAAGTTCTGCAGCACTAA
	Homer1-F	AGGGCTGAACCAACTCAGAA
	Homer1-R	GCAGACGTTCTGCTTCCTCT
	mGluR1-F	TTCCCAAGGCTAGAGTGGTG
	mGluR1-R	TGTCTGCCCATCCATCACTT
	mGluR5-F	GTCAGCTGTTGTTGGACCTG
	mGluR5-R	GGTCACCCCATCGAAGATAC
	GAPDH-F	GAAGGTGAAGGTCGGAGTC
	GAPDH-R	GAAGATGGTGATGGGATTTC
Cloning	*NCDN-Xho1-F*	GATCTCGAGGCCACCATGTCGTGTTGTGACC
	NCDN-EcoR1-R	TGCAGAATTCTGGGCTCTGACAGGCAC
PCR Screening	*NCDN-Scr-F*	CACCACGCTAAGCTCATGTC
	NCDN-Scr-R	GGGGCTGGCTATGTCTACTC
	NCDN-Scr-F2	AGAGCTGCTGTAAAGGGTGT
	NCDN-Scr-R2	TAGAGATGTGAAGCAGGCGT
Internal primers	*NCDN-Scr-F3*	TGGTCCTGCTCCATCTCAAG
	NCDN-Scr-R3	CTGCTGGGAAAGGTCATTGG
	U6-F	GACTATCATATGCTTACCGT
	NCDN-F	AACTCTGTCAAGCCCGAGAT
	acGFP-R	GCTGAACTTGTGGCCATTCA
Off targets	*NCDN-OT1-F*	GGAGACCTTCCCTACCCTGA
	NCDN-OT1-R	TCGACAATAAAGCATTGGAAGA
	NCDN-OT2-F	GCTCCAGACTGGCAGGAGTA
	NCDN-OT2-R	AATGCCTTAATTCATGCAACC

Table S3. Antibodies used in the study.

Antibody	Species	Supplier	Cat. #	Dilution
Anti-ncdn	Rabbit	Sigma Aldrich	#hpa023676	1:1000
Anti-beta-tubulin	Mouse	Sigma Aldrich	#t8660	1:10000
Anti-gaba	Rabbit	Sigma Aldrich	#a2052	1:4000
Anti-phospho-erk	Rabbit	Cell Signaling Technology	#9101	1:1000
Anti-total-erk	Mouse	Cell Signaling Technology	#9107	1:1000
AF555 anti-mouse	Donkey	Thermo Fisher Scientific	#a-31572	1:1000
AF488 goat anti-mouse	Goat	Thermo Fisher Scientific	#a-11001	1:1000
Ird800 anti-rabbit	Donkey	LI-COR	#925-32213	1:5000
IRDye 680LT anti-mouse	Donkey	LI-COR	#925-68022	1:5000

Supplemental methods

Whole exome sequencing (WES)

For family 1 the DNA was sheared using a Covaris instrument (Covaris Inc.), fragment libraries were created using AB Library Builder System (Life Technologies) and size selected on the BluePippin instrument (Sage Science). Target enrichment was performed using Ion AmpliSeq (Life Technologies) according to the manufacturer's protocols. Captured DNA was amplified by emulsion PCR using Ion OneTouch 2 system and Ion PI Template OT2 200 Kit chemistry (Life Technologies), followed by enrichment using Ion OneTouch ES. Samples were loaded on an Ion PI chip and sequenced on Ion Proton System using Ion PI Sequencing 200 Kit (200 bp read length; Life Technologies). Alignment of reads to the human reference sequence (hg19 assembly) and variant detection was performed using v2.1 of the LifeScope™ Software (Life Technologies). Custom R scripts were used to identify potentially damaging variants being shared between the patients whereas absent in homozygous form and present in less than 1% in the other ~1000 exomes in our in-house database, comprising >200 exomes from Pakistani individuals. Variant allele frequencies and missense Z scores was retrieved from the v2 data set of the Genome Aggregation Database (gnomAD; GRCh37/hg19) database consisting of 125,748 exome sequences and 15,708 whole-genome sequences from unrelated individuals. We further removed variants present in homozygous form in the gnomAD database or by segregation analysis. Residual Variation Intolerance Scores (RVIS) was retrieved from the Genic Intolerance Database (genic-intolerance.org) using RVIS release (v4) based on the ExAC v2 variation data. Pathogenicity prediction of identified variants was performed using MutationTaster, Sift, PolyPhen-2 and Combined Annotation Dependent Depletion (CADD) score, the latter three retrieved using Variant Effect Predictor (VEP) from ENSEMBL. For Family 2, the DNA was sheared using a Bioruptor instrument (Diagenode). Library preparation and target enrichment were performed using the Roche MedExome Prep Kit according to manufacturer's recommendations (Roche Diagnostics). Libraries were loaded on an Illumina NextSeq 500 sequencing system (Illumina) and raw reads were mapped to the human genome reference-build

hg19 using the Burrows Wheeler Aligner (BWA MEM v0.717) alignment algorithm. The resulting binary alignment/map (BAM) files were further processed by Genome Analysis Tool Kit HaplotypeCaller (GATK HC v3.8). The VCF files were then annotated on Snpeff version 4.3T. Only coding non-synonymous and splicing variants were considered. Variant prioritization was conducted based on the transmission mode (de novo, autosomal recessive and X-linked), and the frequency of the variants in the gnomAD database. Pathogenicity prediction of identified variants was performed using CADD score. For Family 3 and Family 4, sequencing and analysis were performed as described previously.[1] The genetic tolerance for missense variants in the NCDN protein (UniProt: Q9UBB6) and the specific tolerance at positions for each of the four variant amino acids was predicted by MetaDome (https://stuart.radboudumc.nl/metadome).[2]

Sanger sequencing

Bidirectional Sanger sequencing (Applied Biosystems Big Dye Terminator v3.1 Cycle Sequencing Kit, Applied Biosystems, Life Technologies) was performed on a 3730xl DNA Analyzer (Applied Biosystems, Life Technologies) to confirm candidate gene variants identified from WES and segregation analysis. Sequencher software (Gene Codes Corporation) was used for analysis of Sanger sequencing results.

Generation of *NCDN* knockout clones of SH-SY5Y cells

SH-SY5Y cells were cultured at 37°C and 5% CO2 in 1:1 Dulbecco's modified Eagle's medium (DMEM) and Dulbecco's modified Eagle's medium F12 (DMEM-F12) (Invitrogen) supplemented with 10% FBS (Sigma), 1% GlutaMAXTM™ (Gibco), 100 IU/ml penicillin (pen), and 100 µg/ml streptomycin (strep) (Sigma). Culture medium was changed after every 3-4 days and at 80-90% confluence, cells were passaged using TrypLE™ Express (Gibco).

CRISPR/Cas9 targeting was performed as previously described[3] with slight modifications. In brief, two gRNAs (20 bp each) targeting intron 3 and exon 5 of *NCDN* were selected with the lowest number of predicted off-targets using CRISPR-direct tool (crispr.dbcls.jp). The selected gRNAs were cloned into a pSpCas9(BB)-2A-GFP (PX-458) vector, amplified in One Shot TOP10 Chemically Competent *E.coli* (Thermo Fisher Scientific) and purified using NucleoSpin® Plasmid Kit (Machery-Nagel). CRISPR gRNA plasmid inserts were sequenced at Eurofins (Eurofins Scientific). SH-SY5Y cells were co-transfected with both gRNAs and hygromycin selection vectors (custom selection vector developed for hygromycin selection (unpublished)) for antiobiotic based selection using the jetPRIME transfection reagent (Polyplus). Cells were plated into 96 well plates at low density and selection with hygromycin (300 µg/ml) started twenty-four hours post transfection. Single cell clones were expanded for 2-3 weeks and analyzed for CRISPR/Cas9 mediated gene editing of *NCDN* using Sanger sequencing and PCR-screening primers (Table S1).

Western blot
To obtain whole cell extracts, cells were washed with 1x ice cold PBS and incubated with cold RIPA buffer (Sigma Aldrich) supplemented with Complete, EDTA-free Protease Inhibitor Cocktail (Roche) for 30 min at 4°C on a shaker and centrifuged at 20,000*g* for 20 min at 4°C. Supernatants were collected and protein was quantified using BCA Protein Assay Kit (Thermo Fisher Scientific) according to the manufacturer's instructions. The proteins were denatured in NuPAGE LDS sample buffer (Invitrogen) at 85°C for 10 min, separated on NuPAGE™ 4 to 12% Bis-Tris gels (Invitrogen) under reducing conditions, and blotted onto nitrocellulose membranes using iBLOT system (Invitrogen) according to manufacturer's protocol. Blocking was done using blocking buffer (LI-COR) with 0.1% PBS-T buffer (1:1). Primary antibodies were incubated overnight at 4 °C. The membrane was washed three times with 0.1% PBS-T for 5 min each and incubated with secondary antibodies for 1 h at room temperature. Subsequently, after three washings with 0.1% PBS-T for 5 min each, the signals were visualized on LI-COR Odyssey Platform (LI-COR). Finally, band intensities were quantified using Image Studio version 5.2. Details on antibodies, manufacturers and dilutions used are listed in Table S2.

Isolation of total RNA and qPCR
Total RNA was isolated from fresh SH-SY5H cultures using QIAzol lysis reagent (Qiagen) and RNeasy micro kit (Qiagen) following the manufacturer's guidelines. All extracted RNA samples were quality checked and quantified using NanoDrop™ ND-1000 spectrophotometer (Thermo Fisher Scientific). One µg of total RNA was reverse transcribed (RT-PCR) using High Capacity cDNA Synthesis kit (Thermo Fisher Scientific). Quantitative real-time PCR was performed using SYBR Green Real-Time PCR Master mix (Sigma), on StepOnePlus Real-Time PCR System (Applied Biosystems). The qPCR reactions were performed in triplicates and data was normalized against average expression value of the housekeeping gene *GAPDH*.

Human *NCDN* ORF constructs and site-directed mutagenesis
We obtained a full-length WT human NCDN open reading frame (ORF) entry clone pcDNA3.1-3xFlag-*NCDN* (NovoPro; GenBank: NM_014284.3 and NP_055099.1). The full-length coding sequence of *NCDN* was re-cloned into the mammalian expression vector pAcGFP1-N1 (Clontech) using Xho1 and EcoR1 restriction sites. The four *NCDN* variants NM_014284.3:c.1297G>C, NM_014284.3:c.1433G>A, NM_014284.3:c.1492T>C and NM_014284.3:c.1955C>T were introduced into pAcGFP1-N-*NCDN*-WT expression vectors using QuikChange II Site-Directed Mutagenesis Kit (Agilent technologies Inc.). The primers used for site directed mutagenesis are listed in Table S2. Expression constructs were verified by Sanger sequencing and expressed in SH-SY5Y cells after transfection using jetPRIME (Polyplus).

Neurite outgrowth and neurite number assays

SH-SY5Y cells were seeded on poly-ornithine (PLO) and murine laminin (Sigma Aldrich) coated glass cover slips (16000 cells/cm^2) in 24-well culture plates. After 24 h, differentiation was induced in serum free DMEM/F12 (1:1) medium supplemented with 1 µM retinoic acid (RA; Sigma Aldrich). After seven days of differentiation, cells were fixed using 4% formaldehyde in PBS, washed three times in PBS for 5 minutes each and stained with SimplyBlue™ SafeStain (Thermo Fisher Scientific) for 30 min. Coverslips were mounted with microscope slides using ImmuMount (Thermo Fisher Scientific). The average neurite length per cell was estimated by counting intersections between neurites and test lines of a superimposed frame of fixed size[4] applied on neuronal cultures as described.[5,6] Briefly, 1000-2000 cells were imaged for each group and each per experiment in systematic series of field-views across the whole area of a well using a Zeiss AxioImager microscope AxioImager (Zeiss) at 10x resolution (N.A. 0.45). The number of neurites intersecting with test lines were divided by the total number of soma present in the image and used as a measure of neurite outgrowth. The average number of neurites per cell was manually counted in individual cells from a subset of all collected images using the cell counter plugin of ImageJ software (Fiji version 1.52p).

For transfection and overexpression, KO#9 cells were seeded on PLO and laminin coated 8 mm glass cover slips (24000 cells/cm^2). After 24 h of seeding, cells were transiently transfected with GFP tagged WT or mutant *NCDN*, respectively, or with the empty vector pAcGFP-N1 as a reference for transfection. The jetPRIME transfection reagent (Polyplus) was used for transfection according to the manufacturer's instructions. At 24 hours post-transfection, cells were differentiated, imaged and analyzed as described above.

Electrophysiology

Whole-cell recordings on WT or the NCDN depleted SH-SY5Y cells (KO#2 and KO#9) were performed at room temperature (22-25°C) under an upright microscope (Axon Examiner, Zeiss) after one week of RA induced differentiation. Traces were recorded using an Axoclamp 200B amplifier, filtered at 2 kHz, digitized at 10 kHz with an analog-to-digital converter and analyzed with the pClamp 10.2 software (Molecular Devices). Recording pipettes were pulled from borosilicate glass capillaries (Harvard Apparatus) using a DMZ-Universal puller (Zeitz Instruments) to 5-8 MΩ resistance. Recording solutions were prepared according to Santillo et al., 2014.[7] The intracellular solution contained 130 mM K-Gluconate, 20 mM KCl, 0.3 mM NaGTP, 0.2 mM EGTA, 4.0 mM MgATP, 10 mM HEPES and 10 mM Na-Phosphocreatine (285 mOsm; adjusted pH 7.3). The extracellular solution was composed of 140 mM NaCl, 5 mM KCl, 2 mM $CaCl_2$, 2 mM $MgCl_2$, 10 mM HEPES and 10 mM D-Glucose (300 mOsm; adjusted pH 7.4).

Upon establishment of stable whole-cell configuration, resting membrane potentials were calculated by 3-5 minutes recordings in I=0 mode. Action potentials (AP) were then evoked by 100 ms with 10pA current steps from -30 pA to +140 pA from -70 mV holding potentials. The evoked AP were analyzed using the Clampfit Module of the pCLAMP Software version 10.5

(Molecular devices) and GraphPad Prism. In Figures 3B-D and Figure S6, the rising time refers to the time elapsed between the stimulation and the AP peak; the decay time to the time between the AP peak and the end of the repolarization state; and the amplitude to voltage change in the rising phase of the AP.

ERK phosphorylation assay

KO#9 cells were seeded into six-well plates at a density of 3×10^5 cells per well in 2 mL of DMEM/F12 (1:1) supplemented 1% GlutaMAX (Gibco), 100 IU/mL Pen-Strep and 10% FBS. After 24 h, cells were co-transfected with a construct of a full length human *GRM5* (NM_001143831.2) (Sino Biological; #HG29734-ACG) and a *NCDN* construct containing the variant c.1297G>C, c.1433G>A, c.1492T>C, c.1955C>T or WT *NCDN*, respectively, using the jetPRIME transfection reagent (Polyplus). At 24 h post-transfection, cells were serum-starved overnight and at 48 h post-transfection, cells were washed with PBS and seeded in fresh starvation medium containing 100 µM (RS)-3,5-dihydroxyphenylglycine (DHPG) (R&D systems). Following stimulation for 5 min, medium was removed and cells were washed with PBS and then lysed in 100 µL cold RIPA buffer (Sigma), supplemented with complete, EDTA-free protease inhibitor cocktail (Roche). Protein concentration was measured by BCA protein assay (Pierce) and the phosphorylation of ERK1/2 was determined by western blotting as described above. Primary rabbit polyclonal antibodies against phosphorylated ERK1/2 (Thr202/Tyr204) and total ERK1/2 were used (Cell Signaling Technology). The results were analyzed by Image Studio version 5.2 (LI-COR). The intensity of each band was normalized with total ERK. The average of relative band intensity of four blots from independent experiments were analyzed.

Cell-type-specific expression of *NCDN* in fetal cortex

A previously published single-cell RNA sequencing dataset (UCSC Cell Browser (human cerebral cortex), https://cells.ucsc.edu/) was used to investigate specific gene expression in the developing human cerebral cortex.[8] We performed linear dimensional reduction (pca; npcs = 30) and t-stochastic neighbor embedding (tSNE) clustering (dims = 1:20, tsne.method = Rtsne) of 4261 cells using Seurat 3.[9] We defined three main cell clusters using cell markers from the original paper, namely 'radial glia' expressing the marker *VIM*, 'interneurons' expressing the marker *DLX1,* and 'excitatory neurons' expressing the marker *NEUROD6*.[8] Small clusters, including microglia and astrocytes, were annotated as 'Other' (Figure 3F). Within the excitatory neural cluster we similarly distinguished maturing excitatory neurons (mEN), expressing the marker *GRIN2B,* from the immature excitatory neurons (iEN), expressing the marker *NRP1*.[8]

Supplemental references

1. Sekiguchi, F., Tsurusaki, Y., Okamoto, N., Teik, K.W., Mizuno, S., Suzumura, H., Isidor, B., Ong, W.P., Haniffa, M., White, S.M., et al. (2019). Genetic abnormalities in a large cohort of Coffin–Siris syndrome patients. J. Hum. Genet. *64*, 1173–1186.

2. Wiel, L., Baakman, C., Gilissen, D., Veltman, J.A., Vriend, G., and Gilissen, C. (2019). MetaDome: Pathogenicity analysis of genetic variants through aggregation of homologous human protein domains. Hum. Mutat. *40*, 1030–1038.
3. Fatima, A., Schuster, J., Akram, T., Sobol, M., Hoeber, J., and Dahl, N. (2020). Generation of a human Neurochondrin deficient iPSC line KICRi002-A-3 using CRISPR/Cas9. Stem Cell Res. *44*, 101758.
4. Ronn, L.C.B., Ralets, I., Hartz, B.P., Bech, M., Berezin, A., Berezin, V., Moller, A., and Bock, E. (2000). A simple procedure for quantification of neurite outgrowth based on stereological principles. J Neurosci Methods. *10,* 25-32.
5. Shteinfer-Kuzmine, A., Argueti, S., Gupta, R., Shvil, N., Abu-Hamad, S., Gropper, Y., Hoeber, J., Magrì, A., Messina, A., Kozlova, E.N., et al. (2019). A VDAC1-Derived N-Terminal Peptide Inhibits Mutant SOD1-VDAC1 Interactions and Toxicity in the SOD1 Model of ALS. Front. Cell. Neurosci. *13*, 1–16.
6. Dmytriyeva, O., De Diego Ajenjo, A., Lundø, K., Hertz, H., Rasmussen, K.K., Christiansen, A.T., Klingelhofer, J., Nielsen, A.L., Hoeber, J., Kozlova, E., et al. (2020). Neurotrophic Effects of Vascular Endothelial Growth Factor B and Novel Mimetic Peptides on Neurons from the Central Nervous System. ACS Chem. Neurosci. *11*, 1270–1282.
7. Santillo, S., Moriello, A.S., and Maio, V. Di (2014). Electrophysiological variability in the SH-SY5Y cellular line. Gen. Physiol. Biophys. *33*, 121–129.
8. Nowakowski, T.J., Bhaduri, A., Pollen, A.A., Alvarado, B., Mostajo-Radji, M.A., Di Lullo, E., Haeussler, M., Sandoval-Espinosa, C., Liu, S.J., Velmeshev, D., et al. (2017). Spatiotemporal gene expression trajectories reveal developmental hierarchies of the human cortex. Science *358*, 1318–1323.
9. Butler, A., Hoffman, P., Smibert, P., Papalexi, E., and Satija, R. (2018). Integrating single-cell transcriptomic data across different conditions, technologies, and species. Nat. Biotechnol. *36*, 411–420.
