# Supplementary material for: Monoallelic and bi-allelic variants in NCDN cause neurodevelopmental delay, intellectual disability, and epilepsy
Source: Am J Hum Genet. 2021 Mar 11;108(4):739–48. doi: 10.1016/j.ajhg.2021.02.015 (PMC8059333; doi:10.1016/j.ajhg.2021.02.015)
Supplement: Document S2. Article plus supplemental information [file mmc2.pdf]

Monoallelic and bi-allelic variants in *NCDN* cause neurodevelopmental delay, intellectual disability, and epilepsy

Ambrin Fatima,[1,14] Jan Hoeber,[1,15] Jens Schuster,[1,15] Eriko Koshimizu,[2] Carolina Maya-Gonzalez,[1] Boris Keren,[3] Cyril Mignot,[3] Talia Akram,[1,4] Zafar Ali,[4] Satoko Miyatake,[2,5] Junpei Tanigawa,[6] Takayoshi Koike,[7] Mitsuhiro Kato,[8] Yoshiko Murakami,[9] Uzma Abdullah,[10] Muhammad Akhtar Ali,[11] Rein Fadoul,[1] Loora Laan,[1] Casimiro Castillejo-López,[1] Maarika Liik,[12] Zhe Jin,[13] Bryndis Birnir,[13] Naomichi Matsumoto,[2] Shahid M. Baig,[4,14] Joakim Klar,[1] and Niklas Dahl[1,*]

Summary

Neurochondrin (NCDN) is a cytoplasmatic neural protein of importance for neural growth, glutamate receptor (mGluR) signaling, and synaptic plasticity. Conditional loss of *Ncdn* in mice neural tissue causes depressive-like behaviors, impaired spatial learning, and epileptic seizures. We report on *NCDN* missense variants in six affected individuals with variable degrees of developmental delay, intellectual disability (ID), and seizures. Three siblings were found homozygous for a *NCDN* missense variant, whereas another three unrelated individuals carried different *de novo* missense variants in *NCDN*. We assayed the missense variants for their capability to rescue impaired neurite formation in human neuroblastoma (SH-SY5Y) cells depleted of NCDN. Overexpression of wild-type *NCDN* rescued the neurite-phenotype in contrast to expression of *NCDN* containing the variants of affected individuals. Two missense variants, associated with severe neurodevelopmental features and epilepsy, were unable to restore mGluR5-induced ERK phosphorylation. Electrophysiological analysis of SH-SY5Y cells depleted of NCDN exhibited altered membrane potential and impaired action potentials at repolarization, suggesting NCDN to be required for normal biophysical properties. Using available transcriptome data from human fetal cortex, we show that *NCDN* is highly expressed in maturing excitatory neurons. In combination, our data provide evidence that bi-allelic and *de novo* variants in *NCDN* cause a clinically variable form of neurodevelopmental delay and epilepsy, highlighting a critical role for NCDN in human brain development.

Neurodevelopmental disorders (NDDs) are a clinically heterogeneous group of cognitive, neurological, and neuropsychiatric conditions that manifest during childhood.[1] Although the major fraction of NDDs has a multifactorial origin, identification of the genetic basis for rare Mendelian forms has brought important insights into molecular pathways and functional modules of importance for the formation and homeostasis of the human brain.[2] Furthermore, understanding the precise cause of Mendelian NDDs provides critical information for diagnosis, counselling, and the development of specific treatment options.[3] With the implementation of next-generation sequencing technologies, gene variants in hundreds of genes have been associated with Mendelian NDDs, illustrating an extensive genetic heterogeneity.[4,5] It is now estimated that almost half of all affected individuals with severe NDD have a pathogenic and protein-coding *de novo* variant,[4] whereas inherited and recessive acting variants account for only a small fraction of resolved and affected individuals in outbred populations.[6] Despite the enormous progress over the last decades, it is now generally accepted that many genes and genetic mechanisms associated with neurodevelopmental delay remain to be identified in efforts to improve the diagnostic yield.[7,8]

We report on bi-allelic and *de novo* variants in *NCDN* (MIM: 608458) causing NDD and epilepsy with broad clinical variability. The *NCDN* gene encodes neurochondrin (NCDN), initially named norbin, a leucine-rich cytoplasmic protein widely expressed in developing and adult brain.[9,10] The protein is highly conserved and enriched in α helices but without homologies to known functional domains.[11] Originally identified as a regulator of neurite outgrowth,[12] NCDN is an adaptor protein for G-coupled receptors[13] and modulates metabotropic glutamate

[1]Department of Immunology, Genetics and Pathology, Uppsala University and Science for Life Laboratory, Box 815, 751 08 Uppsala, Sweden; [2]Department of Human Genetics, Yokohama City University Graduate School of Medicine, Yokohama, Kanagawa 236-0004, Japan; [3]Center for Molecular and Chromosomal Genetics APHP, Sorbonne University, Pitié-Salpêtrière Hospital, 47-83 Boulevard de l'Hôpital, 75013 Paris, France; [4]Human Molecular Genetics Laboratory, National Institute for Biotechnology and Genetic Engineering, Faisalabad 38000, Pakistan; [5]Clinical Genetics Department, Yokohama City University Hospital, Yokohama, Kanagawa 236-0004, Japan; [6]Department of Pediatrics, Osaka University Graduate School of Medicine, Osaka 565-0871, Japan; [7]National Epilepsy Center, NHO Shizuoka Institute of Epilepsy and Neurological Disorders, Shizuoka 420-8688, Japan; [8]Department of Pediatrics, Showa University School of Medicine, Tokyo 142-8666, Japan; [9]Research Institute for Microbial Diseases, Osaka University, Osaka 565-0871, Japan; [10]University Institute of Biochemistry and Biotechnology, PMAS-Arid Agriculture University, Rawalpindi 46301, Pakistan; [11]School of Biological Sciences, University of the Punjab, Lahore 54590, Pakistan; [12]Unit of Clinical Neurophysiology, Uppsala University Hospital, 751 85 Uppsala, Sweden; [13]Department of Medical Cell Biology, Biomedical Centre, Uppsala University, 751 08 Uppsala, Sweden
[14]Present address: Department of Biological and Biomedical Sciences, Aga Khan University, Karachi 74000, Pakistan
[15]These authors contributed equally
*Correspondence: niklas.dahl@igp.uu.se
https://doi.org/10.1016/j.ajhg.2021.02.015.
© 2021 The Authors. This is an open access article under the CC BY license (http://creativecommons.org/licenses/by/4.0/).

receptor 5 (mGluR5) signaling of importance for synaptic plasticity in the cerebellum and hippocampus.[14] Mice with conditional depletion of *Ncdn* in the nervous system exhibit epileptic seizures and show spatial learning defects.[15] Furthermore, *Ncdn*−/− mice show impaired neural proliferation and maturation, reduced amounts of mGluR5 on the surface of cortical neurons, and abolished long-term potentiation (LTP),[14,16] a form of neuronal plasticity of importance for learning and memory.[16]

The study was conducted on four families and performed in accordance with the Declaration of Helsinki and after written informed consent from all legal guardians. The study was approved by the regional ethical committee of Uppsala, Sweden; the ethical review board at National Institute for Biotechnology and Genetic Engineering, Faisalabad; and the Committees for Ethical Issues at Showa University School of Medicine, Osaka University Graduate School of Medicine, and Yokohama City University School of Medicine.

We initially identified a consanguineous family of Pakistani origin segregating three siblings presenting with similar degree of neurodevelopmental delay, mild ID, and seizures. The healthy parents are first cousins and without family history of NDD. Whole-exome sequencing (WES) performed on DNA from two of the affected siblings uncovered a homozygous missense variant of unknown significance in *NCDN* (c.1297G>C [p.Glu433Gln] [GenBank: NM_014284.3]). Segregation analysis of the variant confirmed that both parents were heterozygous, whereas all three affected siblings, but not a fourth unaffected sibling, were homozygous (Figure 1A). The c.1297G>C variant is predicted to be pathogenic according to MutationTaster and CADD score but tolerated and benign via Sift and PolyPhen-2, respectively (Table S1). Furthermore, c.1297G is conserved with a phastCons score[17] of 0.884. The expression pattern and known functions of NCDN prompted us to search for additional independent affected individuals with NDD and *NCDN* variants. The identification of disease-causing variants has been improved by development of web-based platforms, enabling researchers to ascertain additional affected individuals with allelic variants and to explore genotype–phenotype correlations. Using GeneMatcher,[18] a part of the Matchmaker exchange project,[19] we identified another three simplex affected individuals of French (F2: II.1), Chinese (F3: II.1), and Japanese (F4:II.1) origin carrying *NCDN* variants (Figure 1A). These three independent affected individuals presented with a variable degree of neurodevelopmental delay and carried unique heterozygous *de novo* variants in *NCDN* (c.1433G>A [p.Arg478Glu], c.1492T>C [p.Trp498Arg], and c.1955C>T [p.Pro652Leu] [GenBank: NM_014284.3 for all three variants]), respectively (Figures 1A–1C). The three missense variants were found to have a phastCons score of 1, they localized in highly conserved regions, and they were pathogenic according to the variant intolerance prediction tools MutationTaster, Sift, and PolyPhen-2 (Table S1). Genetic tolerance for each amino acid (aa) position in NCDN (UniProt: Q9UBB6) was further predicted by MetaDome.[20] The p.Glu433Gln variant is considered "slightly tolerant" (tolerance score = 1.01), while p.Arg478Glu (tolerance score = 0.32), p.Trp498Arg (tolerance score = 0.29), and p.Pro652Leu (tolerance score = 0.4) are considered "intolerant" (Figure 1D). *NCDN* has a missense *Z* score of 3.76 (observed/expected = 0.51 [0.46–0.57]), whereas it has a synonymous *Z* score of 0.73 (o/e = 0.94 [0.83–1.05]), suggesting the gene to be highly intolerant to missense variants. This is supported by a residual variation intolerance score (RVIS) of −1.77, indicating that *NCDN* is among the 4.2% most intolerant human genes to variants.[21]

The clinical features of the three affected individuals with *de novo NCDN* variants overlapped with those of the three affected siblings homozygous for c.1297G>C. The phenotypic findings of the altogether six affected individuals are summarized in Table 1. Neurodevelopmental delay was diagnosed in all affected individuals between ages 1 month and 4 years. Follow-up investigations revealed intellectual disability (ID) that varied from mild to severe. Speech delay was shared by all six affected individuals and developed at age 2–3 years in 5/6 individuals and is still absent in one individual now at 5 years of age. Ability to walk was achieved at age 3–5 years in 3/6 affected individuals. One individual is still unable to walk at age 5 years. Four affected individuals had reached the age of 10 years and showed at that time a capability to read and write corresponding to 2–4 years of delay. Head circumference was reduced (<−2 SD) in 2/6 affected individuals and slightly reduced (<−1 SD) in 4/6 affected individuals. Furthermore, a general growth retardation was found in the three siblings of family 1 (F1:II.1, F1:II.2, and F1:II.3) and in individual F4:II.1. The individual F4:II.1 presented with esotropia, hypertelorism, and epicanthus, but facial dysmorphisms were otherwise absent among the affected individuals. Epilepsy was diagnosed in 5/6 affected individuals and was either generalized (n = 4) or focal (n = 1). The three affected siblings in family 1 had sporadic or febrile seizures, whereas two individuals (F3:II.1 and F4:II.1) had a history of early onset myoclonic encephalopathy. The two affected individuals became seizure free after neurosurgery (F3:II.1) or upon treatment with adrenocorticotropic hormone (ACTH) and Zonisamide (F4:II.1). The electroencephalographic (EEG) patterns showed a slightly pathogenic pattern in the three affected siblings and hypsarrhythmia in affected individuals F3:II.1 and F4:II.1. Magnetic resonance imaging (MRI) performed on three independent affected individuals showed signs of delayed myelination in individual F3:II.1, whereas no overt structural abnormalities were detected in individuals F1:II.1 and F4:II.1.

To clarify the pathophysiological effects of the observed *NCDN* variants, we then generated a neural cell model depleted of NCDN by using the human neuroblastoma line SH-SY5Y. When stimulated with retinoic acid (RA), wild-type (WT) SH-SY5Y cells acquire neuron-like

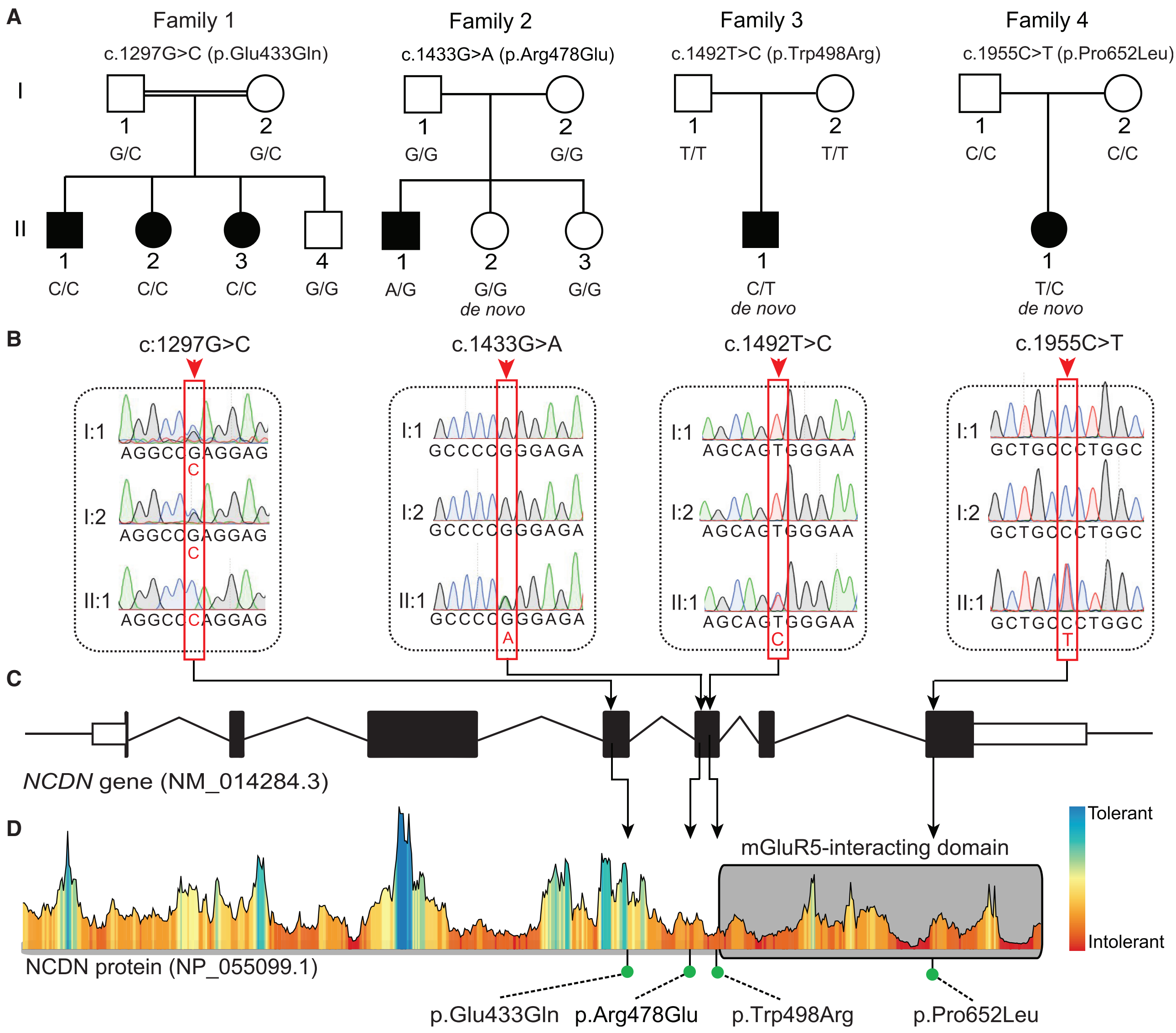

Figure 1. Segregation of rare *NCDN* missense variants in families with neurodevelopmental phenotypes
(A) Pedigrees of four unrelated families with affected members indicated as filled circles (females) and squares (males). Double horizontal lines indicate consanguinity (first cousins).
(B) Representative chromatograms are shown for each *NCDN* variant indicated by vertical red boxes and arrowheads.
(C) Schematic representation of *NCDN* spanning seven exons with relative positions of variants identified in this study. Filled boxed regions denote coding sequences and white boxed regions denote untranslated regions.
(D) Tolerance landscape visualization for missense variants in NCDN via MetaDome[20] with relative positions of the four predicted amino acid (aa) substitutions. The graph indicates that variants close to, or within, the mGluR5-interacting domain (gray box)[14] are less tolerated. The p.Glu433 position, affected by the recessive missense variant c.1297G>C, is "slightly tolerant," while the three heterozygous *de novo* variants are "intolerant" for aa substitutions.

phenotypes such as neurite formation, electrical excitability, and expression of neurotransmitters and neurotransmitter receptors.[22] We targeted *NCDN* in WT SH-SY5Y cells by using CRISPR/Cas9 and expanded two independent clones with distinct homozygous *NCDN* deletions, one spanning 16 bp (c.1458_1473del16 [GenBank: NM_014284.3]; SH-SY5Y$^{NCDN\Delta16/\Delta16}$), assigned KO#2, and one spanning 1,158 bp; (chr1: 36,027,723–36,028,880; SH-SY5Y$^{NCDN\Delta1158/\Delta1158}$), assigned KO#9. The 16 bp deletion (KO#2) is located within exon 5 of *NCDN* and the 1,158 bp deletion (KO#9) spans intron 3 to exon 5 (Figure S1). Both deletions are predicted to cause frameshift and the premature stop codons p.Ala486Alafs*45 and p.Val382Alafs*24, respectively. We also generated a control, assigned "control-Cas9," by transfecting WT SH-SY5Y cells with CRISPR/Cas9 but without gRNA. The top predicted off-target sites were analyzed by Sanger sequencing and revealed WT sequences in the KO#2 and KO#9 clones (Figure S2). Furthermore, depletion of NCDN protein was confirmed in both clones by immunoblot analysis (Figure 2A). Given the effects of NCDN on neurogenesis,[16] we then differentiated the control-Cas9,

Table 1. Summary of clinical findings in six affected individuals with *NCDN* missense variants

Family	Family 1			Family 2	Family 3	Family 4
**Individual**	F1:II.1	F1:II.2	F1:II.3	F2:II.1	F3:II.1	F4:II.1
**Gender**	male	female	female	male	male	female
**Age of observed onset**	4 years	4 years	4 years	2.5 years	1 month	4 months
**Age at most recent examination**	16 years	15 years	14 years	13 years	3 years	5 years
**Ethnicity**	Pakistani	Pakistani	Pakistani	French	Chinese	Japanese
***NCDN* variants NM_014284.3**	c.1297G>C (p.Glu433Gln)	c.1297G>C (p.Glu433Gln)	c.1297G>C (p.Glu433Gln)	c.1433G>A (p.Arg478Glu)	c.1492T>C (p.Trp498Arg)	c.1955C>T (p.Pro652Leu)
**Variant type**	homozygous	homozygous	homozygous	*de novo* heterozygous	*de novo* heterozygous	*de novo* heterozygous
**Body height and age**	160 cm at 16 years (−2.5 SD)	149 cm at 15 years (−2.5 SD)	145 cm at 14 years (−2.5 SD)	152 cm at 11 years (+2.0 SD)	96 cm at 3 years (+0.7 SD)	92 cm at 5 years (−3.2 SD)
**Head circumference and age at measurement**	53 cm at 14 years (−1.1 SD)	50 cm at 13 years (−2.8 SD)	50 cm at 12 years (−2.6 SD)	51 cm at 10 years (−1.5 SD)	47 cm at 3 years (−1.6 SD)	49 cm at 5 years (−0.8 SD)
**Facial dysmorphisms**	no	no	no	no	high arched palate	esotropia, hypertelorism, epicanthus
**Ambulation**	walked at age 3 years, normal ambulation	walked at age 5 years, normal ambulation	walked at age 1 year, normal ambulation	walked at age 14 months, normal ambulation	walked at age 3 years, normal ambulation	unable to walk at age 5 years
**Speech development**	first words at age 2–3 years, normal speech at age 16 years	first words at age 2 years, normal speech at age 15 years	first words at age 2 years, normal speech at age 14 years	first words at age 3 years, profound speech problems at age 13 years	first words at age 2 years	no meaningful words at age 5 years
**Learning disabilities**	read and write with difficulties at age 16 years	read and write with difficulties at age 15 years	read and write with difficulties at age 14 years	read and write like a beginner at age 10 years	N/A	N/A
**Vision, hearing**	normal	normal	normal	normal	normal	normal
**ID**	yes, mild	yes, mild	yes, mild	yes, mild	yes, moderate	yes, severe
**Seizures**	yes	yes (febrile)	yes (febrile)	no	yes	yes
**Frequency**	1–2/month	rare	rare	–	50 times/day prior to treatment	5 times/day prior to treatment
**Type of seizures**	generalized	generalized, fever induced	generalized, fever induced	–	epileptic spasms, focal	epileptic spasms, myoclonus
**Medication**	valproic acid	no medication	valproic acid	–	valproic acid, topiramate, perampanel, and ACTH; seizure-free after surgery	seizure-free with ACTH and ZNS
**Brain MRI**	normal structures	not performed	not performed	not performed	delayed myelination	normal structures
**EEG**	slightly abnormal	slightly abnormal	slightly abnormal	normal at 3 years	hypsarrhythmia at age 4 months	hypsarrhythmia at age 1 year

N/A, not available; ACTH, adrenocorticotropic hormone; ZNS, zonisamide.

KO#2, and KO#9 lines for 7 days with RA, allowing for neurite formation (Figure S3).[22] Image-based analysis revealed that neurites were formed in all three lines (Figure 2B). We then estimated the average neurite length in each line by counting intersections between neurites and test lines of a superimposed frame of fixed size[23] applied on neuronal cultures as described previously.[24] The number of neurites intersecting with test lines were divided by the total number of soma present in the image and used as a measure of neurite outgrowth. The average number of neurites per cell was manually counted in individual cells from a subset of all collected images via the cell counter plugin of ImageJ software (Fiji v.1.52p). We found that the neurites in the two NCDN-deficient lines were shorter and fewer in number when compared with neurites in the control-Cas9 line expressing endogenous NCDN. To clarify whether the

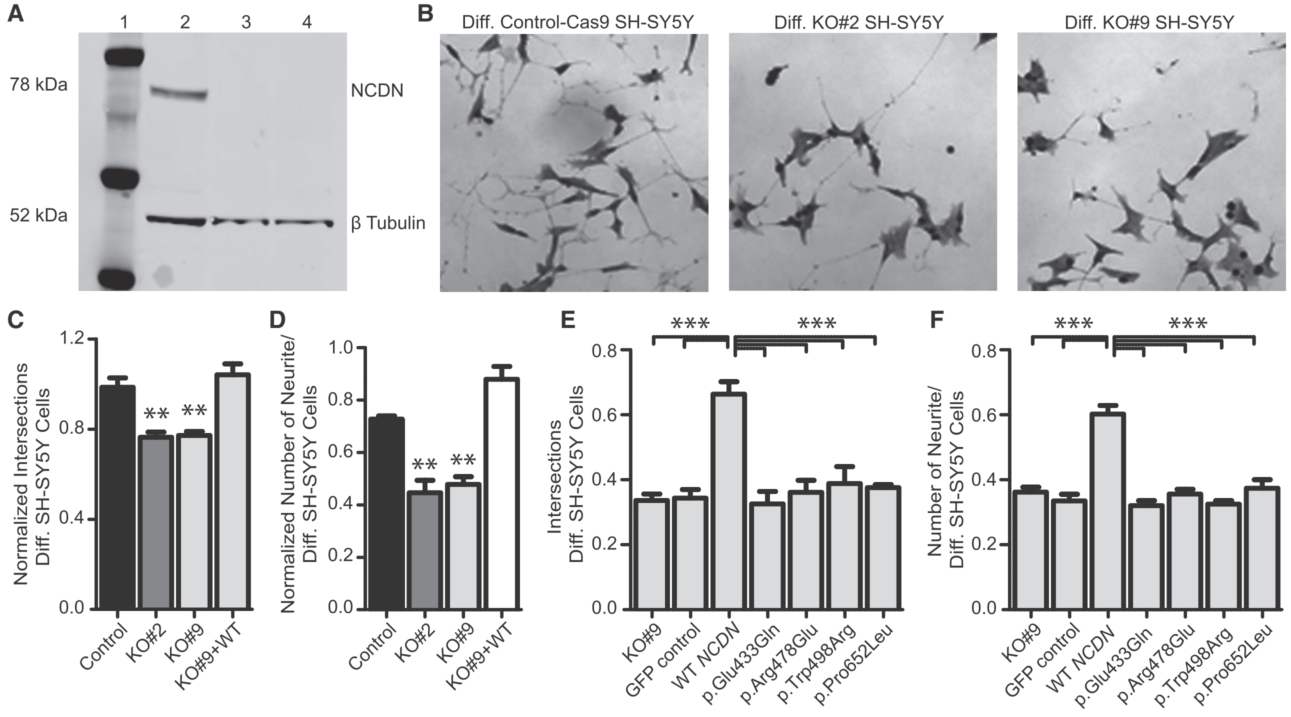

Figure 2. Missense variants in *NCDN* alter length and number of neurites in SH-SY5Y cells

(A) Immunoblot analysis of WT SH-SY5Y (lane 2) and the two independent SH-SY5Y cell clones, KO#2 (lane 3) and KO#9 (lane 4), with CRISPR/Cas9-induced homozygous deletions in *NCDN*, confirming depletion of NCDN protein. An *Anti-Ncdn* antibody (Sigma-Aldrich) was used with β-tubulin (Sigma-Aldrich) as a loading control. A PageRuler Plus pre-stained protein ladder (lane 1) was used as molecular weight markers (Thermo).

(B) Bright field microscopy images of control-Cas9 SH-SY5Y cells (left) and the two independent SH-SY5Y clones, KO#2 and KO#9 (mid and right). Cells were differentiated (Diff.) for one week.

(C) Neurite length in differentiated SH-SY5Y lines. The clones KO#2 and KO#9 show reduced neurite length when compared to the control clone (Cas9-transfected without gRNA). Neurite length was completely rescued in KO#9 after expression of WT *NCDN*. Data were normalized to WT SH-SY5Y cells differentiated in parallel and set to 1.0.

(D) Neurite number in differentiated SH-SY5Y lines. Cells of clones KO#2 and KO#9 showed a reduced neurite number when compared to the Cas9-transfected control clone. Complete restoration of neurite numbers was observed in KO#9 cells after expression of WT *NCDN*. Data were normalized as in (C).

(E) Overexpression of WT *NCDN* and *NCDN* containing the four missense variants in KO#9 SH-SY5Y line. Expression of WT NCDN rescued the neurite length in KO#9. In contrast, overexpression of *NCDN* encoding the missense variants p.Glu433Gln, p.Arg478Glu, p.Trp498Arg, and p.Pro652Leu failed to rescue the reduced neurite length in KO#9. The transfection control (GFP control) showed a neurite outgrowth similar to KO#9.

(F) Expression of WT NCDN rescued the neurite number in KO#9. Overexpression of the four *NCDN* variants in KO#9 failed to rescue the reduced number of neurites. The transfection control (GFP control) showed a neurite number similar to KO#9. The experiments were performed in triplicate and data are shown as mean ± standard error of the mean represented by error bars. Statistical analysis of three independent experiments was performed by one-way ANOVA with Dunnett's multiple-comparisons test (**$p < 0.01$; ***$p < 0.001$).

altered neurite outgrowth was a direct effect of *NCDN* deficiency, we then expressed WT NCDN in the KO#9 clone. Transfection with a full-length WT *NCDN* construct (pAcGFP1-N1-*NCDN*-WT) followed by differentiation revealed a restoration of both length and numbers of neurites in KO#9 (Figures 2C and 2D). We next investigated whether the four *NCDN* missense variants of our affected individuals could restore the neurite length and number in SH-SY5Y cells depleted of *NCDN*. To this end, KO#9 cells were transfected with full-length *NCDN* expression constructs containing any of the four *NCDN* variants: c.1297G>C (p.Glu433Gln), c.1433G>A (p.Arg478Glu), c.1492T>C (p.Trp498Arg), or c.1955C>T (p.Pro652Leu). Transfection with a WT *NCDN* construct was used as reference. At 24 h post-transfection, cells were differentiated for 7 days via RA stimulation. Image-based analysis of KO#9 cells transfected with each of the four variant constructs revealed that both length and number of neurites were consistently reduced when compared with KO#9 cells transfected with WT *NCDN* (Figures 2E and 2F). The four variants thus failed to rescue the neurite phenotypes that were comparable to non-transfected KO#9 cells. Furthermore, neurite formation in KO#9 cells transfected with pAcGFP1-N1-empty vector were similar to non-transfected KO#9 cells (Figures 2E and 2F). These data suggest that the impaired neurite formation in SH-SY5Y cells depleted of NCDN is rescued by the expression of WT NCDN but not by *NCDN* constructs expressing the four missense variants.

NCDN interacts with mGluR1 and mGluR5, encoded by *GRM1* (MIM: 604473) and *GRM5* (MIM: 604102).[14,25]

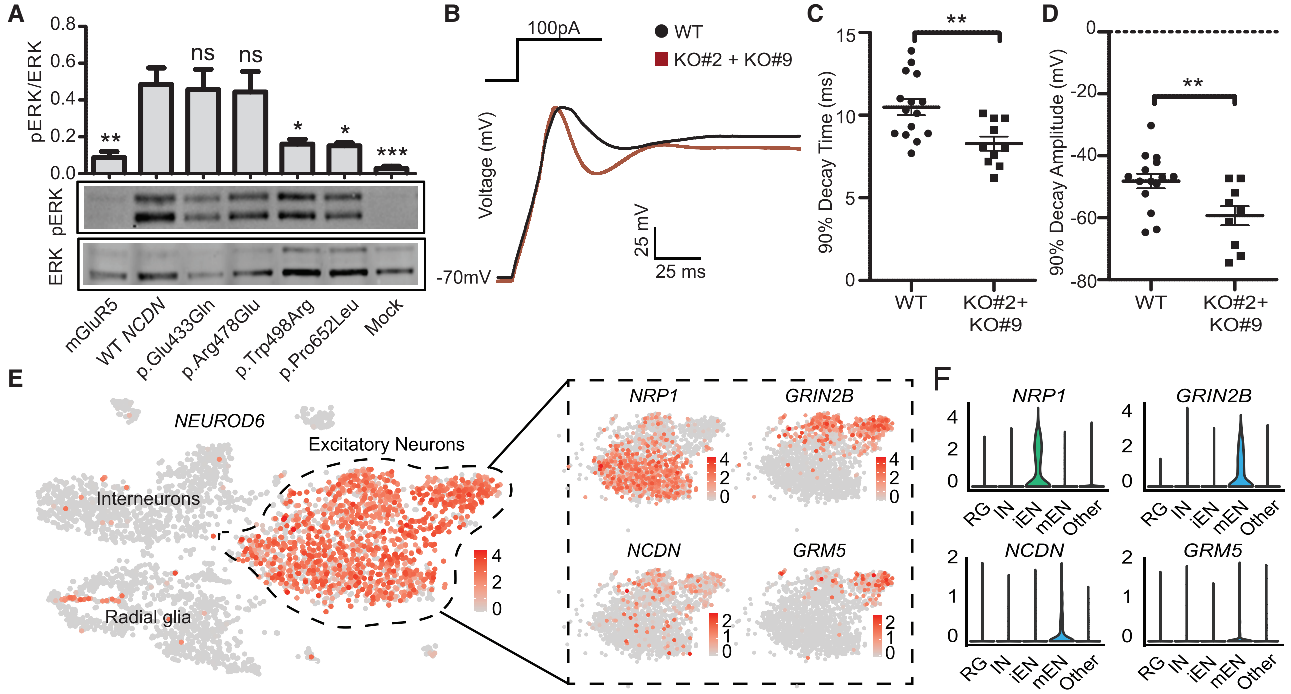

Figure 3. *NCDN* variants interfere with mGluR5 signaling and alter electrophysiological properties of SH-SY5Y cells

(A) ERK1/2 phosphorylation in transfected KO#9 cells expressing WT NCDN or NCDN containing each of the p.Glu433Gln, p.Arg478-Glu, p.Trp498Arg, and p.Pro652Leu variants when compared to cells expressing only mGluR5. DHPG-induced extracellular signal-regulated kinase (ERK) phosphorylation was quantified in serum-starved transfected KO#9 cells expressing the mGluR5 receptor alone, mGluR5 receptor co-expressed with WT NCDN, or co-expressed with NCDN containing p.Glu433Gln, p.Arg478Glu, p.Trp498Arg, and p.Pro652Leu. The p.Glu433Gln and p.Arg478Glu rescued phosphorylation to normal levels, whereas p.Trp498Arg and p.Pro652Leu, positioned in the mGluR5 interacting domain, did not. The phosphorylation of ERK1/2 was determined by immunoblot and band intensities correspond to phospho-ERK1/2 and total ERK1/2. Phosphorylated ERK1/2 was normalized with total ERK1/2. Data are shown as mean ± standard error of the mean represented by error bars. Statistical analysis of four independent experiments was performed by one-way ANOVA with Dunnett's multiple-comparisons test (*$p < 0.05$; **$p < 0.01$; ***$p < 0.001$; ns = not significant). Mock = untransfected.

Representative immunoblot (n = 5, bottom panels) of DHPG-induced ERK phosphorylation.

(B) Abortive action potentials (APs) evoked in response to rectangular current injections in differentiated SH-SY5Y cells with a holding potential of −70 mV and current injections of 100 pA for 100 ms, suggesting different decay kinetics between NCDN-depleted (KO#2 [n = 5], KO#9 [n = 5]) and WT (n = 15) SH-SY5Y cells. The average rising time was similar when comparing KO#2 and KO#9 (32.18 ± 0.74 ms) with WT SH-SY5Y (33.35 ± 0.64 ms) cells at injected currents of 100 pA. The average voltage responses show abortive AP.

(C and D) 90% AP decay time (C) and 90% AP decay amplitude (D) show a significant change in AP decay kinetics in KO#2 and KO#9 SH-SY5Y cells when compared to WT SH-SY5Y cells. Data are shown as mean ± standard error of the mean represented by error bars. Statistical analysis was carried out with unpaired t tests with Welch correction (**$p < 0.005$). mV, millivolts; ms, milliseconds.

(E) Cell-type-specific expression of *NCDN* in the human cortex derived from fetuses at post-conceptional weeks 6–37. The t-SNE plot shows the excitatory neural cell cluster defined by *NEUROD6* expression (left panel). The cluster is further divided into immature excitatory neurons (iENs), expressing *NRP1,* and maturing excitatory neurons (mENs), expressing *GRIN2B* (boxed). Expression of *NCDN* and *GRM5* are detected mainly at the mEN stage. Expression levels are scaled, log-normalized data from Seurat 3. Expression levels are color-coded in the feature plots.

(F) Expression levels of the five genes in the excitatory cell cluster of fetal cortex showing enriched expression of *NCDN* and *GRM5* in mEN. RG, radial glia; IN, interneurons; iEN, immature excitatory neurons; mEN, maturing excitatory neurons.

Activation of mGluR5 induces the phosphorylation of extracellular signal-regulated kinase (ERK)[26] that can be used as a measure of mGluR activity.[27] We therefore reasoned that the effect of *NCDN* variants on mGluR1/5-induced signaling can be assessed by quantification of ERK phosphorylation. To this end, GFP-tagged WT *NCDN* and each of the four mutant constructs were co-transfected with a full-length *GRM5* construct into KO#9 cells. The *GRM5* construct transfected into KO#9 cells was used as control. Transfection efficiency was confirmed by fluorescence after 24 h, followed by serum starvation for 24 h. Activation of mGluRs via dihydroxyphenylglycine (DHPG) was induced, followed by cell harvest. Immunoblot analysis of protein lysates from the cells revealed a significant increase in ERK1/2 phosphorylation in cells expressing WT *NCDN* when compared with cells expressing only *GRM5* (Figure 3A). Compared with WT *NCDN*, the overexpression of *NCDN* constructs containing the c.1492T>C or c.1955C>T variants showed markedly reduced levels of phosphorylated ERK1/2 when assessed by immunoblotting. This observation is consistent with the position of the two missense variants in a region of *NCDN* encoding the mGluR5-interacting domain (Figures 1D and 3A).[14,28] In contrast, overexpression of the *NCDN*

variants c.1297G>C and c.1433G>A, replacing residues outside the mGluR5-interacting domain of NCDN, showed no apparent effect on ERK1/2 phosphorylation (Figure 1A). Besides the interaction with mGluR5, NCDN tethers or mediates the activation of several proteins important for downstream signaling through phosphorylation of both ERK and CaMKII.[14,15,29] We therefore sought to investigate the effect of loss of NCDN in SH-SY5Y on the interacting partners mGluR1 and mGluR5, as well as on calmodulin-dependent kinase II (CaMKII). Using qPCR, we observed differential expression for the three NCDN-interacting partners when comparing *NCDN*-deficient and WT SH-SY5Y cells (Figure S4). Our findings suggest that the two variants c.1492T>C and c.1955C>T, affecting residues in the mGluR-interacting domain, inhibit mGluR1/5-induced ERK phosphorylation and that NCDN depletion in SH-SY5Y cells results in altered expression of factors belonging to the mGluR pathway.

Seizure was a shared feature in 5/6 affected individuals in our study. Furthermore, mice with a homozygous conditional disruption of *Ncdn* in the central nervous system exhibit epilepsy.[15] We therefore set out to investigate the electrophysiological properties of *NCDN*-deficient SH-SY5Y cells by using whole-cell patch-clamp recordings. Cultures of KO#9 and WT SH-SY5Y lines were differentiated for 7 days. Cell sizes from both cultures were similar when evaluated by capacitance measurements (Figure S5A). The patch-clamp analysis revealed a resting membrane hyperpolarization in KO#2 and KO#9 lines (-45.00 ± 0.55 mV) when compared to WT SH-SY5Y (-38.23 mV $\pm$ 1.58 mV; Figure S5B). Upon 100 ms rectangular current injections, NCDN-depleted lines showed abortive action potentials (APs) and without differences in the rising phase of the APs (Figure 3B). However, AP repolarization kinetics was significantly different, and there was an increased repolarization rate (mV/ms) in *NCDN*-deficient cells in response to different injected currents (Figures 3C, 3D, and S6). These observations strongly suggest altered electrophysiological properties in *NCDN*-deficient and differentiated SH-SY5Y cells, consistent with seizures in mice with conditional depletion of *Ncdn*.

We then sought to investigate the expression pattern of *NCDN* in human fetal cortex by using available and previously published data.[30] In this data, we observed that *NCDN* expression is enriched in excitatory neural cells defined by *NEUROD6* (MIM: 611513) expression (Figure 3E). Specifically, the expression of *NCDN* is predominantly found in maturing excitatory neurons (mENs), defined by expression of the glutamate ionotropic receptor gene *GRIN2B* (MIM:138252), but not in immature excitatory neurons (iENs), defined by *NRP1* (MIM: 602069) expression (Figure 3F).[30] Similarly, analysis of *GRM5* expression showed expression levels confined to the mEN stage (Figures 3E and 3F). These data show that *NCDN* is highly expressed in mENs of the fetal cortex together with *GRM5*, consistent with interactions between NCDN and mGluR5.

Our series of six affected individuals with *NCDN* variants shared several core NDD features such as learning disability and ID, delayed speech development, and variable degrees of reduced head circumference. While all four *NCDN* variants failed to rescue the impaired neurite formation in NCDN-depleted SH-SY5Y cells, the inheritance, onset, severity, and combination of symptoms varied between families without shared distinctive clinical features among affected individuals. The three siblings (F1:II.1, F1:II.2, and F1:II.3) with a bi-allelic *NCDN* variant c.1297G>C presented with a uniform phenotype with growth retardation, mild ID, and sporadic or febrile seizures. Their heterozygous parents had neither neurological symptoms nor cognitive impairment. Individual F2:II.1, carrying a heterozygous *de novo* variant c.1433G>A, showed a similar mild ID and speech delay but no seizures. On the other hand, individuals F3:II.1 and F4:II.1 with the *de novo* variants c.1492T>C and c.1955C>T, respectively, showed a more severe form of developmental delay with an earlier onset, moderate to severe ID, and epileptic spasms in our series of patients. Interestingly, *in vitro* expression of the c.1492T>C and c.1955C>T variants, situated in the mGluR5-interacting domain,[14,28] interfered with mGluR5-induced ERK phosphorylation in our assay. On the basis of a combination of our clinical observations and experimental data, it is thus tempting to speculate that the c.1297G>C and c.1433G>A variants, without ability to promote neurite formation and with no detectable effects on ERK phosphorylation in our assays, alter NCDN functions in slightly different ways than the c.1492T>C and c.1955C>T variants, showing a reduced mGluR5-mediated ERK phosphorylation (Figure 3A). In a prior report, three individuals were identified with heterozygous *de novo* deletions spanning 1.1 Mb to 3.1 Mb involving *NCDN*.[31] The three affected individuals presented with mild to moderate ID and motor and speech delay. However, seizures were not reported, suggesting that *NCDN* haploinsufficiency does not necessarily cause epilepsy. It may therefore be hypothesized that the seizures, associated with three missense variants in our study, define a sub-phenotype mediated by a dominant-negative effect. The variant *NCDN* allele c.1297G>C in family 1 should in such a case be hypomorphic to explain the silent phenotype in heterozygous parents. The nucleotide position c.1297G has a lower phastCons score (0.884) and the corresponding aa residue is more tolerated (MetaDome tolerance score) when compared with the aa residues corresponding to the three positions c.1433G, c.1492T, and c.1955C, consistent with hypomorphism as a plausible explanation. Domain-specific missense variants leading to alternate mechanisms and phenotypic pleiotropy have recently been reported. Specific bi-allelic variants in the transcription factor gene *DEAF1* (MIM: 602635) appear to cause hypomorphic loss-of-function alleles, whereas some heterozygous *de novo* variants have a dominant-negative (antimorph) effect.[32] In *TRIO* (MIM: 609701), encoding a guanine nucleotide exchange factor, domain-specific missense variants mediate either haploinsufficiency or gain of function leading to

distinct phenotypes.[33] Similar to DEAF1 and TRIO, NCDN interacts with multiple proteins and impacts different functions of the central nervous system.[13,34,35] We therefore suggest that the four different missense variants may cause distinct and domain-specific changes of NCDN functions, ultimately leading to the phenotypic variability. A detailed understanding on how the identified *NCDN* variants interfere with binding to different protein partners will now require further investigations.

In conclusion, we identified four different missense variants in *NCDN* that are functionally relevant in six individuals presenting with a variable degree of neurodevelopmental delay, ID, and seizures. Five out of the six affected individuals were diagnosed with variable types of epilepsy, a core phenotypic feature of NDD with a 10-fold increased prevalence in individuals with ID compared with the general population.[36] Three *NCDN* variants occurred *de novo*, whereas one variant segregated in a bi-allelic state in three siblings with a uniform clinical presentation. Functional characterization of the four *NCDN* variants showed that they, in contrast to WT *NCDN*, were unable to rescue the impaired neurite formation in NCDN-depleted SH-SY5Y cells. Furthermore, mGluR signaling, measured by ERK phosphorylation levels, was compromised by *NCDN* variants located in the mGluR5-binding domain of the protein in two individuals presenting with severe clinical features of NDD. Despite the functional effects shown for the four *NCDN* variants in our assays, the precise mechanisms leading to the variable clinical presentations and different inheritance patterns remain elusive. Mice with conditional loss of *Ncdn* in the central nervous system exhibit learning defects and epilepsy. These features are concordant with the phenotype in five of our affected individuals, however they are not mediated by complete loss of function. Furthermore, large heterozygous deletions in patients with NDD suggest NCDN haploinsufficiency is not associated with epilepsy. However, a dominant-negative effect, in particular of the c.1297G>C and c.1433G>A variants, is plausible and would require the recessive variant c.1297G>C to be hypomorphic. Given that NCDN interacts with multiple partners in the central nervous system during development and that the *NCDN* variants identified may affect these interactions differently, we therefore hypothesize that the variants interfere with NCDN functions in different ways, ultimately leading to variable clinical features. However, the precise mechanistic explanations for the variable molecular, cellular, and clinical effects of the monoallelic and bi-allelic *NCDN* variants warrant further investigations, requiring mapping of functional domains of NCDN and its 3D structure.

Data and code availability

The *NCDN* variants with accession numbers are available at https://www.ncbi.nlm.nih.gov/clinvar/ (GenBank: NM_014284.3) for variants c.1297G>C: SCV001443681, c.1433G>A: SCV001443682, c.1492T>C: SCV001443683, and c.1955C>T: SCV001443684. WES datasets have not been deposited in a public repository because of privacy and ethical restrictions but are available from the corresponding author on request.

Supplemental information

Supplemental information can be found online at https://doi.org/10.1016/j.ajhg.2021.02.015.

Acknowledgments

We thank the study participants and their parents for cooperation. The exomes of family F1 were generated at the Genome platform, Science for Life Laboratory, and cell images were obtained at the BioVis platform, Uppsala University, Sweden. Computations were performed on resources provided by SNIC through Uppsala Multidisciplinary Center for Advanced Computational Science (UPPMAX), Uppsala University, Sweden. This work was supported by grants from the Swedish Research Council (2015-02424 and 2020-01947) to N.D.; Hjärnfonden (FO2019-0210 and FO2020-0171) to N.D.; Science for Life Laboratory to N.D.; Stiftelsen Margarethahemmet to N.D.; Sävstaholm Society to J.H.; Märta Lundqvist's fund to J.H.; O.E. and E. Johansson's fund to J.H.; Borgström Foundation to A.F.; Japan Agency for Medical Research and Development (AMED; JP20ek0109280, JP20dm0107090, JP20ek0109301, JP20ek0109348, and JP20kk0205012) to N.M. and (JP20lk0201069) to M.K.; JSPS KAKENHI (JP17H0153) to N.M., (JP17K10080) to S.M., (JP19K1692) to E.K., and (JP20K08236) to M.K.; intramural research grants for Neurological and Psychiatric Disorders of NCNP from the Ministry of Health, Labour and Welfare (30-6) to N.M. and M.K. and (30-7) to N.M.; and The Takeda Science Foundation to N.M. The funders played no role in study design, data collection and interpretation, or decision to publish.

Declaration of interests

The authors declare no competing interests.

Received: November 26, 2020
Accepted: February 21, 2021
Published: March 11, 2021

Web resources

CRISPR direct, https://crispr.dbcls.jp/
GeneMatcher, https://genematcher.org/
Genic Intolerance Database, http://genic-intolerance.org/
GnomAD, https://gnomad.broadinstitute.org/
MetaDome, https://stuart.radboudumc.nl/metadome
MutationTaster, http://www.mutationtaster.org/
OMIM, https://omim.org/
Primer 3 (v.0.4.0), https://bioinfo.ut.ee/primer3-0.4.0/
The National Center for Biotechnology Information (NCBI), https://www.ncbi.nlm.nih.gov/
UCSC Cell Browser (human cerebral cortex), https://cells.ucsc.edu/
Variant Effect Predictor (VEP) from ENSEMBL, https://www.ensembl.org/

References

1. Thapar, A., Cooper, M., and Rutter, M. (2017). Neurodevelopmental disorders. Lancet Psychiatry *4*, 339–346.
2. Vissers, L.E.L.M., Gilissen, C., and Veltman, J.A. (2016). Genetic studies in intellectual disability and related disorders. Nat. Rev. Genet. *17*, 9–18.
3. Tărlungeanu, D.C., and Novarino, G. (2018). Genomics in neurodevelopmental disorders: an avenue to personalized medicine. Exp. Mol. Med. *50*, 100.
4. McRae, J.F., Clayton, S., Fitzgerald, T.W., Kaplanis, J., Prigmore, E., Rajan, D., Sifrim, A., Aitken, S., Akawi, N., Alvi, M., et al.; Deciphering Developmental Disorders Study (2017). Prevalence and architecture of de novo mutations in developmental disorders. Nature *542*, 433–438.
5. Coe, B.P., Stessman, H.A.F., Sulovari, A., Geisheker, M.R., Bakken, T.E., Lake, A.M., Dougherty, J.D., Lein, E.S., Hormozdiari, F., Bernier, R.A., and Eichler, E.E. (2019). Neurodevelopmental disease genes implicated by de novo mutation and copy number variation morbidity. Nat. Genet. *51*, 106–116.
6. Martin, H.C., Jones, W.D., McIntyre, R., Sanchez-Andrade, G., Sanderson, M., Stephenson, J.D., Jones, C.P., Handsaker, J., Gallone, G., Bruntraeger, M., et al.; Deciphering Developmental Disorders Study (2018). Quantifying the contribution of recessive coding variation to developmental disorders. Science *362*, 1161–1164.
7. Short, P.J., McRae, J.F., Gallone, G., Sifrim, A., Won, H., Geschwind, D.H., Wright, C.F., Firth, H.V., FitzPatrick, D.R., Barrett, J.C., and Hurles, M.E. (2018). De novo mutations in regulatory elements in neurodevelopmental disorders. Nature *555*, 611–616.
8. Kaplanis, J., Samocha, K.E., Wiel, L., Zhang, Z., Arvai, K.J., Eberhardt, R.Y., Gallone, G., Lelieveld, S.H., Martin, H.C., McRae, J.F., et al.; Deciphering Developmental Disorders Study (2020). Evidence for 28 genetic disorders discovered by combining healthcare and research data. Nature *586*, 757–762.
9. Shinozaki, K., Kume, H., Kuzume, H., Obata, K., and Maruyama, K. (1999). Norbin, a neurite-outgrowth-related protein, is a cytosolic protein localized in the somatodendritic region of neurons and distributed prominently in dendritic outgrowth in Purkinje cells. Brain Res. Mol. Brain Res. *71*, 364–368.
10. Xu, Y., Li, Z., Yao, L., Zhang, X., Gan, D., Jiang, M., Wang, N., Chen, G., and Wang, X. (2017). Altered Norbin Expression in Patients with Epilepsy and a Rat Model. Sci. Rep. *7*, 13970.
11. Wang, H., Nong, Y., Bazan, F., Greengard, P., and Flajolet, M. (2010). Norbin: A promising central nervous system regulator. Commun. Integr. Biol. *3*, 487–490.
12. Shinozaki, K., Maruyama, K., Kume, H., Kuzume, H., and Obata, K. (1997). A novel brain gene, norbin, induced by treatment of tetraethylammonium in rat hippocampal slice and accompanied with neurite-outgrowth in neuro 2a cells. Biochem. Biophys. Res. Commun. *240*, 766–771.
13. Pan, D., Barber, M.A., Hornigold, K., Baker, M.J., Toth, J.M., Oxley, D., and Welch, H.C.E. (2016). Norbin stimulates the catalytic activity and plasma membrane localization of the guanine-nucleotide exchange factor P-Rex1. J. Biol. Chem. *291*, 6359–6375.
14. Wang, H., Westin, L., Nong, Y., Birnbaum, S., Bendor, J., Brismar, H., Nestler, E., Aperia, A., Flajolet, M., and Greengard, P. (2009). Norbin is an endogenous regulator of metabotropic glutamate receptor 5 signaling. Science *326*, 1554–1557.
15. Dateki, M., Horii, T., Kasuya, Y., Mochizuki, R., Nagao, Y., Ishida, J., Sugiyama, F., Tanimoto, K., Yagami, K., Imai, H., and Fukamizu, A. (2005). Neurochondrin negatively regulates CaMKII phosphorylation, and nervous system-specific gene disruption results in epileptic seizure. J. Biol. Chem. *280*, 20503–20508.
16. Wang, H., Warner-Schmidt, J., Varela, S., Enikolopov, G., Greengard, P., and Flajolet, M. (2015). Norbin ablation results in defective adult hippocampal neurogenesis and depressive-like behavior in mice. Proc. Natl. Acad. Sci. USA *112*, 9745–9750.
17. Siepel, A., Bejerano, G., Pedersen, J.S., Hinrichs, A.S., Hou, M., Rosenbloom, K., Clawson, H., Spieth, J., Hillier, L.W., Richards, S., et al. (2005). Evolutionarily conserved elements in vertebrate, insect, worm, and yeast genomes. Genome Res. *15*, 1034–1050.
18. Sobreira, N., Schiettecatte, F., Valle, D., and Hamosh, A. (2015). GeneMatcher: a matching tool for connecting investigators with an interest in the same gene. Hum. Mutat. *36*, 928–930.
19. Philippakis, A.A., Azzariti, D.R., Beltran, S., Brookes, A.J., Brownstein, C.A., Brudno, M., Brunner, H.G., Buske, O.J., Carey, K., Doll, C., et al. (2015). The Matchmaker Exchange: a platform for rare disease gene discovery. Hum. Mutat. *36*, 915–921.
20. Wiel, L., Baakman, C., Gilissen, D., Veltman, J.A., Vriend, G., and Gilissen, C. (2019). MetaDome: Pathogenicity analysis of genetic variants through aggregation of homologous human protein domains. Hum. Mutat. *40*, 1030–1038.
21. Petrovski, S., Wang, Q., Heinzen, E.L., Allen, A.S., and Goldstein, D.B. (2013). Genic intolerance to functional variation and the interpretation of personal genomes. PLoS Genet. *9*, e1003709.
22. Santillo, S., Schiano Moriello, A., and Di Maio, V. (2014). Electrophysiological variability in the SH-SY5Y cellular line. Gen. Physiol. Biophys. *33*, 121–129.
23. Rønn, L.C.B., Ralets, I., Hartz, B.P., Bech, M., Berezin, A., Berezin, V., Møller, A., and Bock, E. (2000). A simple procedure for quantification of neurite outgrowth based on stereological principles. J. Neurosci. Methods *100*, 25–32.
24. Shteinfer-Kuzmine, A., Argueti, S., Gupta, R., Shvil, N., Abu-Hamad, S., Gropper, Y., Hoeber, J., Magrì, A., Messina, A., Kozlova, E.N., et al. (2019). A VDAC1-Derived N-Terminal Peptide Inhibits Mutant SOD1-VDAC1 Interactions and Toxicity in the SOD1 Model of ALS. Front. Cell. Neurosci. *13*, 346.
25. Lu, Y.M., Jia, Z., Janus, C., Henderson, J.T., Gerlai, R., Wojtowicz, J.M., and Roder, J.C. (1997). Mice lacking metabotropic glutamate receptor 5 show impaired learning and reduced CA1 long-term potentiation (LTP) but normal CA3 LTP. J. Neurosci. *17*, 5196–5205.
26. Hu, H.J., Alter, B.J., Carrasquillo, Y., Qiu, C.S., and Gereau, R.W., 4th. (2007). Metabotropic glutamate receptor 5 modulates nociceptive plasticity via extracellular signal-regulated kinase-Kv4.2 signaling in spinal cord dorsal horn neurons. J. Neurosci. *27*, 13181–13191.
27. Uematsu, K., Heiman, M., Zelenina, M., Padovan, J., Chait, B.T., Aperia, A., Nishi, A., and Greengard, P. (2015). Protein kinase A directly phosphorylates metabotropic glutamate receptor 5 to modulate its function. J. Neurochem. *132*, 677–686.
28. Sergin, I., Jong, Y.I., Harmon, S.K., Kumar, V., and O'Malley, K.L. (2017). Sequences within the C terminus of the metabotropic glutamate receptor 5 (mGluR5) are responsible for inner nuclear membrane localization. J. Biol. Chem. *292*, 3637–3655.

29. Kalinowska, M., and Francesconi, A. (2016). Group I Metabotropic Glutamate Receptor Interacting Proteins: Fine-Tuning Receptor Functions in Health and Disease. Curr. Neuropharmacol. *14*, 494–503.
30. Nowakowski, T.J., Bhaduri, A., Pollen, A.A., Alvarado, B., Mostajo-Radji, M.A., Di Lullo, E., Haeussler, M., Sandoval-Espinosa, C., Liu, S.J., Velmeshev, D., et al. (2017). Spatiotemporal gene expression trajectories reveal developmental hierarchies of the human cortex. Science *358*, 1318–1323.
31. Tokita, M.J., Chow, P.M., Mirzaa, G., Dikow, N., Maas, B., Isidor, B., Le Caignec, C., Penney, L.S., Mazzotta, G., Bernardini, L., et al. (2015). Five children with deletions of 1p34.3 encompassing AGO1 and AGO3. Eur. J. Hum. Genet. *23*, 761–765.
32. Nabais Sá, M.J., Jensik, P.J., McGee, S.R., Parker, M.J., Lahiri, N., McNeil, E.P., Kroes, H.Y., Hagerman, R.J., Harrison, R.E., Montgomery, T., et al. (2019). De novo and biallelic DEAF1 variants cause a phenotypic spectrum. Genet. Med. *21*, 2059–2069.
33. Barbosa, S., Greville-Heygate, S., Bonnet, M., Godwin, A., Fagotto-Kaufmann, C., Kajava, A.V., Laouteouet, D., Mawby, R., Wai, H.A., Dingemans, A.J.M., et al.; C4RCD Research Group (2020). Opposite Modulation of RAC1 by Mutations in TRIO Is Associated with Distinct, Domain-Specific Neurodevelopmental Disorders. Am. J. Hum. Genet. *106*, 338–355.
34. Ohoka, Y., Hirotani, M., Sugimoto, H., Fujioka, S., Furuyama, T., and Inagaki, S. (2001). Semaphorin 4C, a transmembrane semaphorin, [corrected] associates with a neurite-outgrowth-related protein, SFAP75. Biochem. Biophys. Res. Commun. *280*, 237–243.
35. Francke, F., Ward, R.J., Jenkins, L., Kellett, E., Richter, D., Milligan, G., and Bächner, D. (2006). Interaction of neurochondrin with the melanin-concentrating hormone receptor 1 interferes with G protein-coupled signal transduction but not agonist-mediated internalization. J. Biol. Chem. *281*, 32496–32507.
36. McGrother, C.W., Bhaumik, S., Thorp, C.F., Hauck, A., Branford, D., and Watson, J.M. (2006). Epilepsy in adults with intellectual disabilities: prevalence, associations and service implications. Seizure *15*, 376–386.

The American Journal of Human Genetics, Volume *108*

Supplemental information

Monoallelic and bi-allelic variants in *NCDN* cause neurodevelopmental delay, intellectual disability, and epilepsy

Ambrin Fatima, Jan Hoeber, Jens Schuster, Eriko Koshimizu, Carolina Maya-Gonzalez, Boris Keren, Cyril Mignot, Talia Akram, Zafar Ali, Satoko Miyatake, Junpei Tanigawa, Takayoshi Koike, Mitsuhiro Kato, Yoshiko Murakami, Uzma Abdullah, Muhammad Akhtar Ali, Rein Fadoul, Loora Laan, Casimiro Castillejo-López, Maarika Liik, Zhe Jin, Bryndis Birnir, Naomichi Matsumoto, Shahid M. Baig, Joakim Klar, and Niklas Dahl

Supplemental case report

Family 1 (F1:II.1, F1:II.2 and F1:II.3) was recruited from National Institute for Biotechnology and Genetic Engineering, Faisalabad, Pakistan (S.M.). Additional cases were enrolled from Pitié-Salpêtrière Hospital, Paris, France (C.M. and B.K., case F2:II.1), and Yokohama City University Graduate School of Medicine, Japan (E.K. and N.M., cases F3:II.1 and F4:II.1). Venous blood samples were obtained from participants and genomic DNA was extracted according to standard protocols. All participating research centers were connected through the public data sharing platform GeneMatcher (entry "NCDN").

Supplemental figures

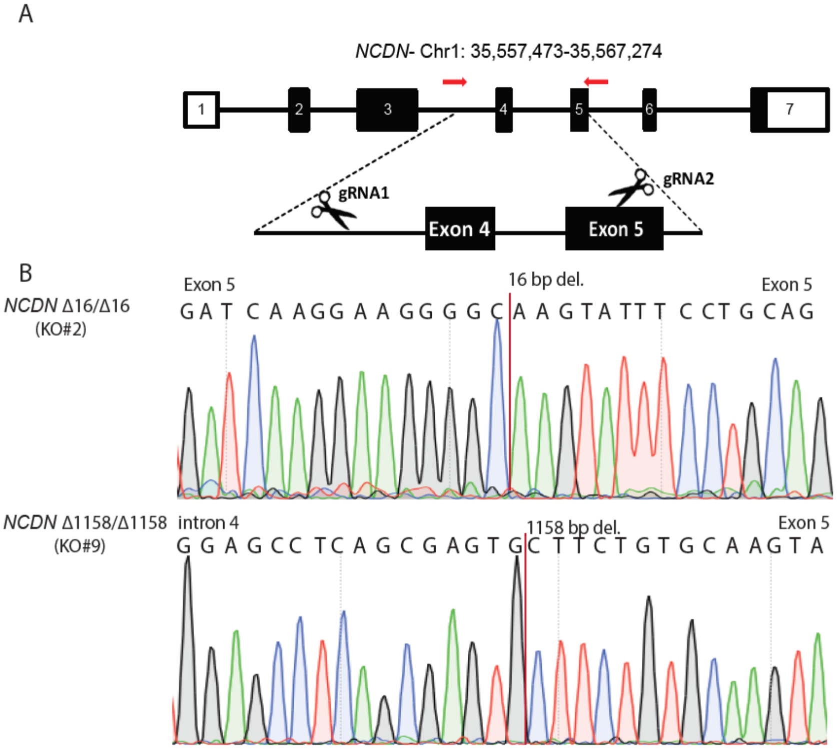

Figure S1. **Schematic presentation of CRISPR/Cas9 targeting of *NCDN* in SH-SY5Y cells.** (A) Relative positions of *NCDN* guide RNAs (gRNAs) designed to target intron 3 and exon 5 of the *NCDN* gene. Screening of SH-SY5Y cell clones edited for *NCDN* was carried out using PCR primers flanking both gRNAs (red arrows) and in-between both gRNAs (not shown) in different combinations. (B) Sanger sequencing of *NCDN* clones derived from single cells revealed a homozygous deletion of 16 bp in exon 5 (clone *NCDN*Δ16/Δ16, assigned KO#2), and a 1158 bp deletion spanning intron 3 to exon 5 (clone *NCDN*Δ1158/Δ1158, assigned KO#9).

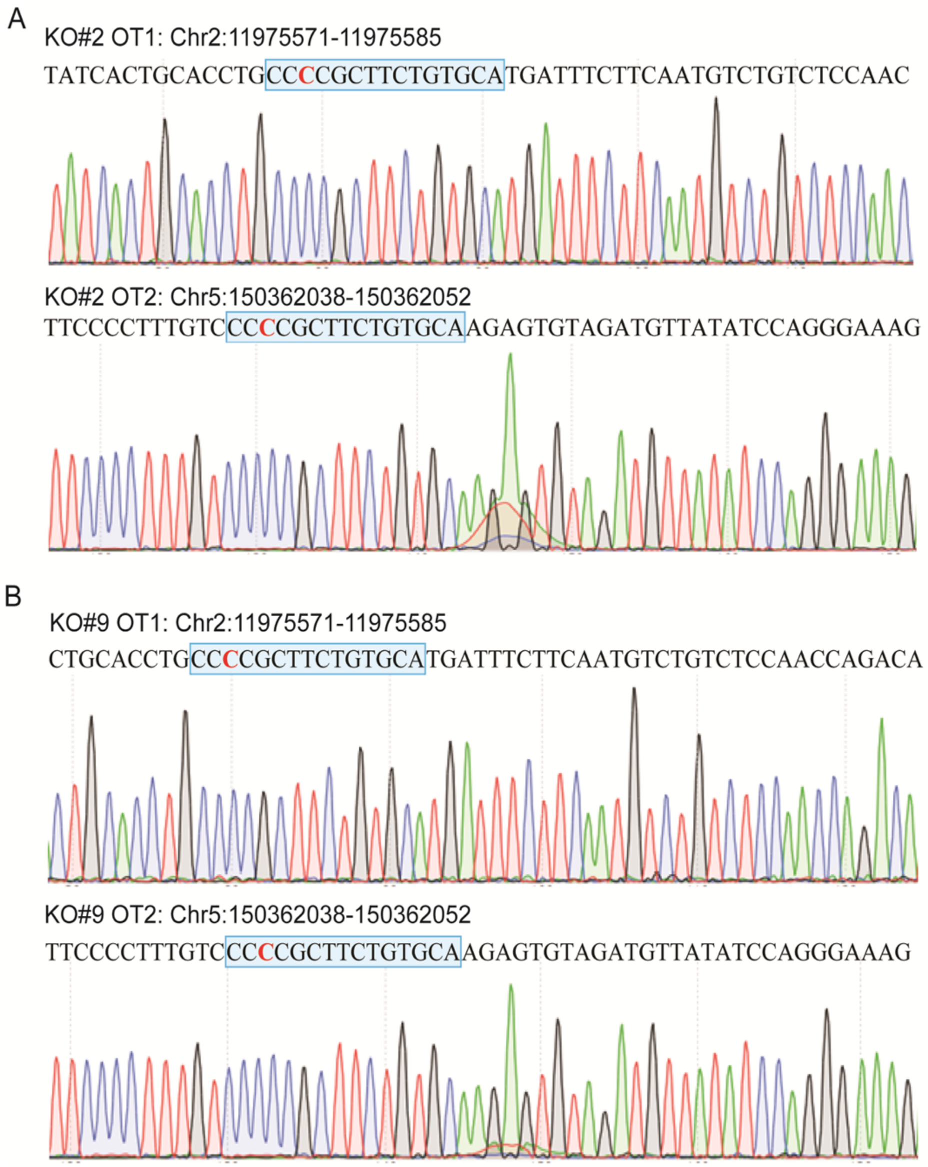

Figure S2. Analysis of potential off-target sites by Sanger sequencing in SH-SY5Y clones KO#2 and KO#9.

Sequence chromatogram of the two top-predicted off targets (*OT1* and *OT2*) in (A) KO#2 cells and (B) KO#9 cells. Matched sequences are highlighted in blue and mismatch nucleotides are marked in red.

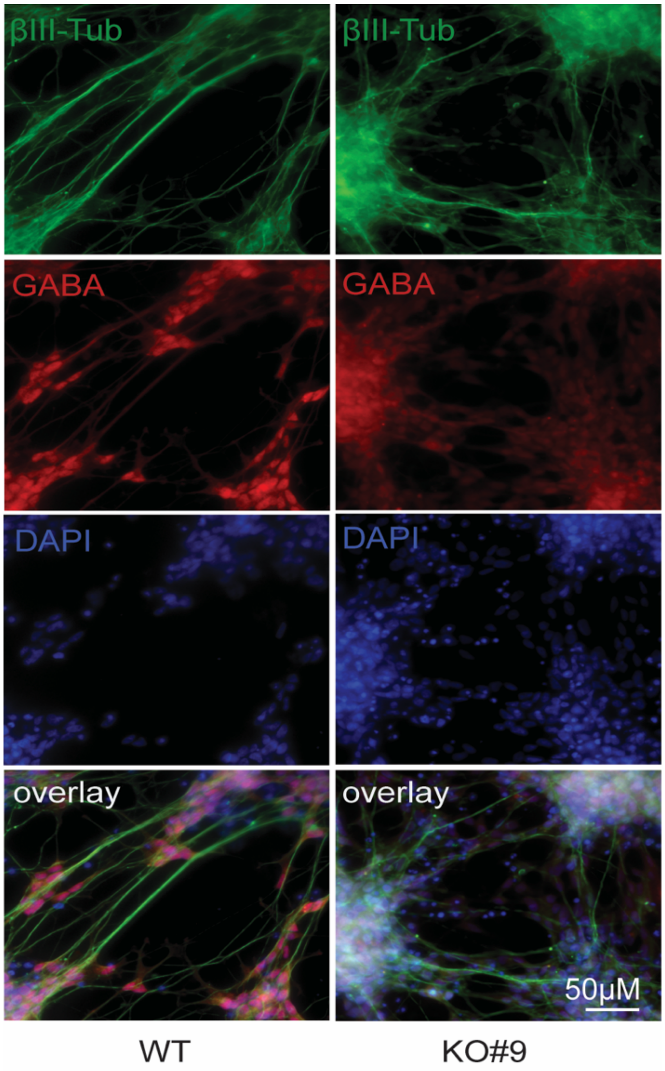

Figure S3. Differentiation and staining of SH-SY5Y cells.
Representative images of WT and KO#9 SH-SY5Y cells differentiated for seven days and stained for βIII-Tubulin (green) and GABA (red). Cells are counterstained with the nuclear marker DAPI (blue). Stainings are merged at the bottom. Size bar: 50 µm.

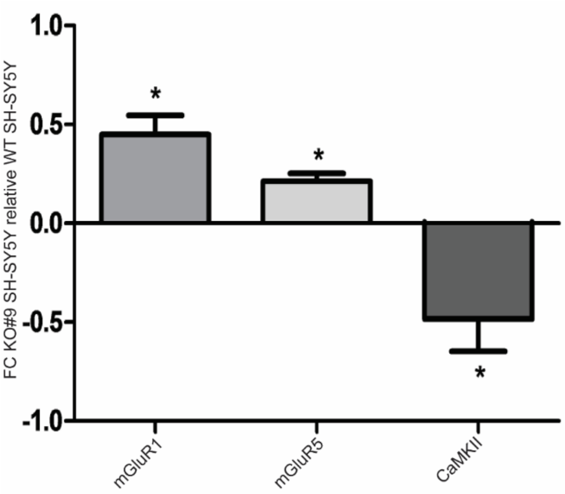

Figure S4. Depletion of NCDN in SH-SY5Y cells alters expression of genes encoding proteins in the group I mGluR pathway.

Expression of genes encoding the NCDN interacting partners mGluR1, mGluR5 and Calmodulin Dependent Kinase II (CaMKII), analyzed by RT-qPCR in un-differentiated KO#9 cells. Expression levels are presented as fold change (FC) values corresponding to $\log_2(2^{-\Delta\Delta C_T})$ after normalization to GAPDH in WT SH-SY5Y. A FC of zero indicates similar expression in WT SH-SY5Y and KO#9. Data was obtained from three biological replicates. Error bars correspond to SEM. Statistical analysis was carried out with unpaired Students t-tests with Welch's correction (*$p < 0.05$).

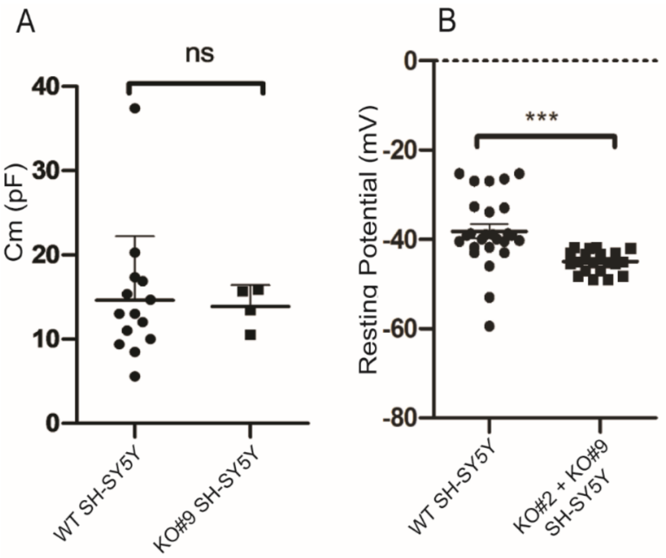

Figure S5. Passive membrane properties of WT SH-SY5Y and NCDN deficient SH-SY5Y cells from patch-clamp measurements.

(A) Membrane capacitance, as a measurement of cell surface area, of WT SH-SY5Y (n=14) and KO#9 SH-SY5Y cells (n=4). Cells were differentiated for one week with RA before being measured. The results indicate similar sizes for WT and NCDN deficient SH-SH5Y cells. (B) Resting membrane potential in cells differentiated for one week with RA. Significant resting membrane hyperpolarization is observed in the NCDN deficient SH-SY5Y cells KO#2 and KO#9 (n=20) when compared to WT SH-SH5Y (n=26). Error bars represent SEM; Cm: cell capacitance; pF: picofarads; mV: millivolts. Statistical analysis was carried out using unpaired t-tests with Welch correction (***$p<0.0005$).

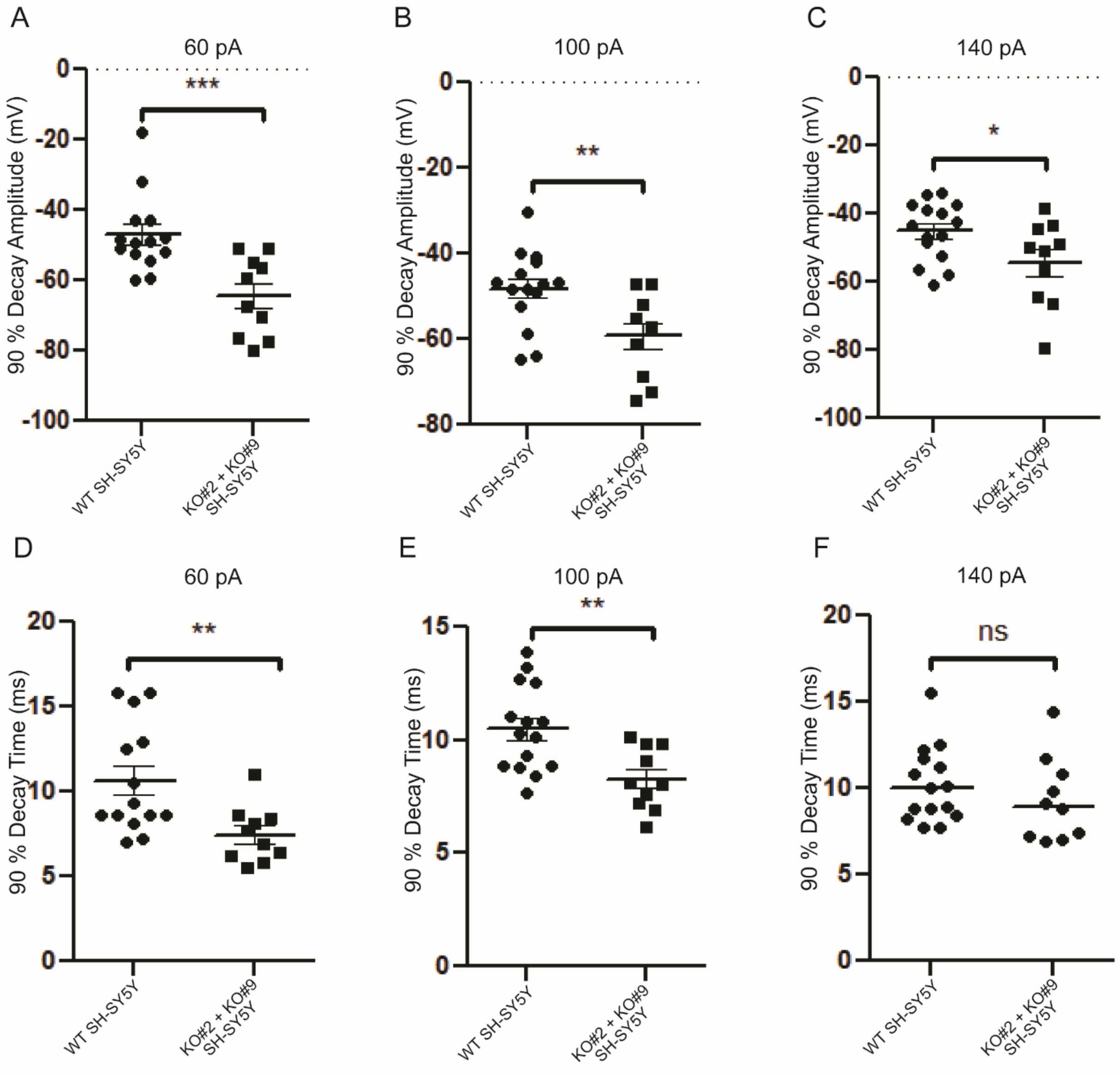

Figure S6. **Kinetics of action potential repolarization.**
Whole-cell patch clamp recordings of WT SH-SY5Y (n=14) and NCDN deficient SH-SY5Y cells (KO#2: n=5 and KO#9: n=5) after stimulus-dependent impulses. Cells were stimulated with rectangular currents of (A) 60 pA (B) 100 pA and (C) 140 pA for 100 ms at a holding potential of -70 mV. Action potential decay amplitude was reduced in NCDN deficient SH-SY5Y cells using all three conditions. (D-F) Action potential decay time was found reduced in NCDN deficient SH-SY5Y cells at (D) 60 pA (E) 100 pA and (F) 140 pA. The decay amplitude corresponds to the voltage change of the rising phase of the AP and the decay time defines the time between the AP-peak and end of repolarization state. Error bars represent SEM. Statistical analyses were carried out by unpaired t-tests with Welch correction (*$p<0.05$; **$p<0.005$; ***$p<0.0005$).

Supplemental tables

Table S1. Pathogenicity prediction of *NCDN* (NM_014284.3) variants by different *in silico* tools.

Variation (hg19)	Mutation Taster	Sift	Sift Score	PolyPhen-2	HumVar	PhyloP	PhastCons	CADD
1:36028146;c.1297G>C	disease causing	tolerated	0.57	benign	0.216	2.158	0.884	21.5
1:36028850;c.1433G>A	disease causing	damaging	0	probably damaging	0.997	5.029	1	28.8
1:36028909:c.1492T>C	disease causing	damaging	0	probably damaging	0.997	1.569	1	26.0
1:36031029:c.1955C>T	disease causing	damaging	0.05	possibly damaging	0.766	3.530	1	24.5

Table S2. Primers used in this study.

Methods	Primer name	5' – 3' sequence
CRISPR-Cas9	*NCDN-grna1-Top*	CACCGCTGGAGCCTCAGCGAGTACG
	NCDN-grna1-Bottom	AAACCGTACTCGCTGAGGCTCCAGC
	NCDN-grna2-Top	CACCgCCTCGCTTCTGTGCAAGTAT
	NCDN-grna2-Bottom	AAACATACTTGCACAGAAGCGAGGc
Site directed mutagen.	*NCDN-p.E433Q-F*	CTACGAGGAGGCCCAGGAGGCCAATGA
	NCDN-p.E433Q-R	TCATTGGCCTCCTGGGCCTCCTCGTAG
	NCDN-p.R478E-F	GAAGATGGGCCCCAGGAGATCCTGATCA
	NCDN-p.R478E-R	TGATCAGGATCTCCTGGGGCCCATCTTC
	NCDN-p.W498R-F	TATTTCCTGCAGCAGCGGGAACTCACATCCC
	NCDN-p.W498R-R	GGGATGTGAGTTCCCGCTGCTGCAGGAAATA
	NCDN- p.P652L -F	GTGTGCCTCTGCTGCTCTGGCTGGCCCCCG
	NCDN- p.P652L -R	CGGGGGCCAGCCAGAGCAGCAGAGGCACAC
	NCDN- p.P652L -R	CGGGGGCCAGCCAGAGCAGCAGAGGCACAC
qPCR	*CAMKIIB-F*	TGTCAGCCAGAGATCACCAG
	CAMKIIB-R	ACTCTCTCGCCACAATGTCT
	CaM2-F	GGCACAATTGACTTCCCTGA
	CaM2-R	GCGAAGTTCTGCAGCACTAA
	Homer1-F	AGGGCTGAACCAACTCAGAA
	Homer1-R	GCAGACGTTCTGCTTCCTCT
	mGluR1-F	TTCCCAAGGCTAGAGTGGTG
	mGluR1-R	TGTCTGCCCATCCATCACTT
	mGluR5-F	GTCAGCTGTTGTTGGACCTG
	mGluR5-R	GGTCACCCCATCGAAGATAC
	GAPDH-F	GAAGGTGAAGGTCGGAGTC
	GAPDH-R	GAAGATGGTGATGGGATTTC
Cloning	*NCDN-Xho1-F*	GATCTCGAGGCCACCATGTCGTGTTGTGACC
	NCDN-EcoR1-R	TGCAGAATTCTGGGCTCTGACAGGCAC
PCR Screening	*NCDN-Scr-F*	CACCACGCTAAGCTCATGTC
	NCDN-Scr-R	GGGGCTGGCTATGTCTACTC
	NCDN-Scr-F2	AGAGCTGCTGTAAAGGGTGT
	NCDN-Scr-R2	TAGAGATGTGAAGCAGGCGT
Internal primers	*NCDN-Scr-F3*	TGGTCCTGCTCCATCTCAAG
	NCDN-Scr-R3	CTGCTGGGAAAGGTCATTGG
	U6-F	GACTATCATATGCTTACCGT
	NCDN-F	AACTCTGTCAAGCCCGAGAT
	acGFP-R	GCTGAACTTGTGGCCATTCA
Off targets	*NCDN-OT1-F*	GGAGACCTTCCCTACCCTGA
	NCDN-OT1-R	TCGACAATAAAGCATTGGAAGA
	NCDN-OT2-F	GCTCCAGACTGGCAGGAGTA
	NCDN-OT2-R	AATGCCTTAATTCATGCAACC

Table S3. Antibodies used in the study.

Antibody	Species	Supplier	Cat. #	Dilution
Anti-ncdn	Rabbit	Sigma Aldrich	#hpa023676	1:1000
Anti-beta-tubulin	Mouse	Sigma Aldrich	#t8660	1:10000
Anti-gaba	Rabbit	Sigma Aldrich	#a2052	1:4000
Anti-phospho-erk	Rabbit	Cell Signaling Technology	#9101	1:1000
Anti-total-erk	Mouse	Cell Signaling Technology	#9107	1:1000
AF555 anti-mouse	Donkey	Thermo Fisher Scientific	#a-31572	1:1000
AF488 goat anti-mouse	Goat	Thermo Fisher Scientific	#a-11001	1:1000
Ird800 anti-rabbit	Donkey	LI-COR	#925-32213	1:5000
IRDye 680LT anti-mouse	Donkey	LI-COR	#925-68022	1:5000

Supplemental methods

Whole exome sequencing (WES)

For family 1 the DNA was sheared using a Covaris instrument (Covaris Inc.), fragment libraries were created using AB Library Builder System (Life Technologies) and size selected on the BluePippin instrument (Sage Science). Target enrichment was performed using Ion AmpliSeq (Life Technologies) according to the manufacturer's protocols. Captured DNA was amplified by emulsion PCR using Ion OneTouch 2 system and Ion PI Template OT2 200 Kit chemistry (Life Technologies), followed by enrichment using Ion OneTouch ES. Samples were loaded on an Ion PI chip and sequenced on Ion Proton System using Ion PI Sequencing 200 Kit (200 bp read length; Life Technologies). Alignment of reads to the human reference sequence (hg19 assembly) and variant detection was performed using v2.1 of the LifeScope™ Software (Life Technologies). Custom R scripts were used to identify potentially damaging variants being shared between the patients whereas absent in homozygous form and present in less than 1% in the other ~1000 exomes in our in-house database, comprising >200 exomes from Pakistani individuals. Variant allele frequencies and missense Z scores was retrieved from the v2 data set of the Genome Aggregation Database (gnomAD; GRCh37/hg19) database consisting of 125,748 exome sequences and 15,708 whole-genome sequences from unrelated individuals. We further removed variants present in homozygous form in the gnomAD database or by segregation analysis. Residual Variation Intolerance Scores (RVIS) was retrieved from the Genic Intolerance Database (genic-intolerance.org) using RVIS release (v4) based on the ExAC v2 variation data. Pathogenicity prediction of identified variants was performed using MutationTaster, Sift, PolyPhen-2 and Combined Annotation Dependent Depletion (CADD) score, the latter three retrieved using Variant Effect Predictor (VEP) from ENSEMBL. For Family 2, the DNA was sheared using a Bioruptor instrument (Diagenode). Library preparation and target enrichment were performed using the Roche MedExome Prep Kit according to manufacturer's recommendations (Roche Diagnostics). Libraries were loaded on an Illumina NextSeq 500 sequencing system (Illumina) and raw reads were mapped to the human genome reference-build

hg19 using the Burrows Wheeler Aligner (BWA MEM v0.717) alignment algorithm. The resulting binary alignment/map (BAM) files were further processed by Genome Analysis Tool Kit HaplotypeCaller (GATK HC v3.8). The VCF files were then annotated on Snpeff version 4.3T. Only coding non-synonymous and splicing variants were considered. Variant prioritization was conducted based on the transmission mode (de novo, autosomal recessive and X-linked), and the frequency of the variants in the gnomAD database. Pathogenicity prediction of identified variants was performed using CADD score. For Family 3 and Family 4, sequencing and analysis were performed as described previously.[1] The genetic tolerance for missense variants in the NCDN protein (UniProt: Q9UBB6) and the specific tolerance at positions for each of the four variant amino acids was predicted by MetaDome (https://stuart.radboudumc.nl/metadome).[2]

Sanger sequencing

Bidirectional Sanger sequencing (Applied Biosystems Big Dye Terminator v3.1 Cycle Sequencing Kit, Applied Biosystems, Life Technologies) was performed on a 3730xl DNA Analyzer (Applied Biosystems, Life Technologies) to confirm candidate gene variants identified from WES and segregation analysis. Sequencher software (Gene Codes Corporation) was used for analysis of Sanger sequencing results.

Generation of *NCDN* knockout clones of SH-SY5Y cells

SH-SY5Y cells were cultured at 37°C and 5% CO2 in 1:1 Dulbecco's modified Eagle's medium (DMEM) and Dulbecco's modified Eagle's medium F12 (DMEM-F12) (Invitrogen) supplemented with 10% FBS (Sigma), 1% GlutaMAXTM™ (Gibco), 100 IU/ml penicillin (pen), and 100 µg/ml streptomycin (strep) (Sigma). Culture medium was changed after every 3-4 days and at 80-90% confluence, cells were passaged using TrypLE™ Express (Gibco).

CRISPR/Cas9 targeting was performed as previously described[3] with slight modifications. In brief, two gRNAs (20 bp each) targeting intron 3 and exon 5 of *NCDN* were selected with the lowest number of predicted off-targets using CRISPR-direct tool (crispr.dbcls.jp). The selected gRNAs were cloned into a pSpCas9(BB)-2A-GFP (PX-458) vector, amplified in One Shot TOP10 Chemically Competent *E.coli* (Thermo Fisher Scientific) and purified using NucleoSpin® Plasmid Kit (Machery-Nagel). CRISPR gRNA plasmid inserts were sequenced at Eurofins (Eurofins Scientific). SH-SY5Y cells were co-transfected with both gRNAs and hygromycin selection vectors (custom selection vector developed for hygromycin selection (unpublished)) for antiobiotic based selection using the jetPRIME transfection reagent (Polyplus). Cells were plated into 96 well plates at low density and selection with hygromycin (300 µg/ml) started twenty-four hours post transfection. Single cell clones were expanded for 2-3 weeks and analyzed for CRISPR/Cas9 mediated gene editing of *NCDN* using Sanger sequencing and PCR-screening primers (Table S1).

Western blot

To obtain whole cell extracts, cells were washed with 1x ice cold PBS and incubated with cold RIPA buffer (Sigma Aldrich) supplemented with Complete, EDTA-free Protease Inhibitor Cocktail (Roche) for 30 min at 4°C on a shaker and centrifuged at 20,000*g* for 20 min at 4°C. Supernatants were collected and protein was quantified using BCA Protein Assay Kit (Thermo Fisher Scientific) according to the manufacturer's instructions. The proteins were denatured in NuPAGE LDS sample buffer (Invitrogen) at 85°C for 10 min, separated on NuPAGE™ 4 to 12% Bis-Tris gels (Invitrogen) under reducing conditions, and blotted onto nitrocellulose membranes using iBLOT system (Invitrogen) according to manufacturer's protocol. Blocking was done using blocking buffer (LI-COR) with 0.1% PBS-T buffer (1:1). Primary antibodies were incubated overnight at 4 °C. The membrane was washed three times with 0.1% PBS-T for 5 min each and incubated with secondary antibodies for 1 h at room temperature. Subsequently, after three washings with 0.1% PBS-T for 5 min each, the signals were visualized on LI-COR Odyssey Platform (LI-COR). Finally, band intensities were quantified using Image Studio version 5.2. Details on antibodies, manufacturers and dilutions used are listed in Table S2.

Isolation of total RNA and qPCR

Total RNA was isolated from fresh SH-SY5H cultures using QIAzol lysis reagent (Qiagen) and RNeasy micro kit (Qiagen) following the manufacturer's guidelines. All extracted RNA samples were quality checked and quantified using NanoDrop™ ND-1000 spectrophotometer (Thermo Fisher Scientific). One µg of total RNA was reverse transcribed (RT-PCR) using High Capacity cDNA Synthesis kit (Thermo Fisher Scientific). Quantitative real-time PCR was performed using SYBR Green Real-Time PCR Master mix (Sigma), on StepOnePlus Real-Time PCR System (Applied Biosystems). The qPCR reactions were performed in triplicates and data was normalized against average expression value of the housekeeping gene *GAPDH*.

Human *NCDN* ORF constructs and site-directed mutagenesis

We obtained a full-length WT human NCDN open reading frame (ORF) entry clone pcDNA3.1-3xFlag-*NCDN* (NovoPro; GenBank: NM_014284.3 and NP_055099.1). The full-length coding sequence of *NCDN* was re-cloned into the mammalian expression vector pAcGFP1-N1 (Clontech) using Xho1 and EcoR1 restriction sites. The four *NCDN* variants NM_014284.3:c.1297G>C, NM_014284.3:c.1433G>A, NM_014284.3:c.1492T>C and NM_014284.3:c.1955C>T were introduced into pAcGFP1-N-*NCDN*-WT expression vectors using QuikChange II Site-Directed Mutagenesis Kit (Agilent technologies Inc.). The primers used for site directed mutagenesis are listed in Table S2. Expression constructs were verified by Sanger sequencing and expressed in SH-SY5Y cells after transfection using jetPRIME (Polyplus).

Neurite outgrowth and neurite number assays

SH-SY5Y cells were seeded on poly-ornithine (PLO) and murine laminin (Sigma Aldrich) coated glass cover slips (16000 cells/cm^2) in 24-well culture plates. After 24 h, differentiation was induced in serum free DMEM/F12 (1:1) medium supplemented with 1 μM retinoic acid (RA; Sigma Aldrich). After seven days of differentiation, cells were fixed using 4% formaldehyde in PBS, washed three times in PBS for 5 minutes each and stained with SimplyBlue™ SafeStain (Thermo Fisher Scientific) for 30 min. Coverslips were mounted with microscope slides using ImmuMount (Thermo Fisher Scientific). The average neurite length per cell was estimated by counting intersections between neurites and test lines of a superimposed frame of fixed size[4] applied on neuronal cultures as described.[5,6] Briefly, 1000-2000 cells were imaged for each group and each per experiment in systematic series of field-views across the whole area of a well using a Zeiss AxioImager microscope AxioImager (Zeiss) at 10x resolution (N.A. 0.45). The number of neurites intersecting with test lines were divided by the total number of soma present in the image and used as a measure of neurite outgrowth. The average number of neurites per cell was manually counted in individual cells from a subset of all collected images using the cell counter plugin of ImageJ software (Fiji version 1.52p).

For transfection and overexpression, KO#9 cells were seeded on PLO and laminin coated 8 mm glass cover slips (24000 cells/cm^2). After 24 h of seeding, cells were transiently transfected with GFP tagged WT or mutant *NCDN*, respectively, or with the empty vector pAcGFP-N1 as a reference for transfection. The jetPRIME transfection reagent (Polyplus) was used for transfection according to the manufacturer's instructions. At 24 hours post-transfection, cells were differentiated, imaged and analyzed as described above.

Electrophysiology

Whole-cell recordings on WT or the NCDN depleted SH-SY5Y cells (KO#2 and KO#9) were performed at room temperature (22-25°C) under an upright microscope (Axon Examiner, Zeiss) after one week of RA induced differentiation. Traces were recorded using an Axoclamp 200B amplifier, filtered at 2 kHz, digitized at 10 kHz with an analog-to-digital converter and analyzed with the pClamp 10.2 software (Molecular Devices). Recording pipettes were pulled from borosilicate glass capillaries (Harvard Apparatus) using a DMZ-Universal puller (Zeitz Instruments) to 5-8 MΩ resistance. Recording solutions were prepared according to Santillo et al., 2014.[7] The intracellular solution contained 130 mM K-Gluconate, 20 mM KCl, 0.3 mM NaGTP, 0.2 mM EGTA, 4.0 mM MgATP, 10 mM HEPES and 10 mM Na-Phosphocreatine (285 mOsm; adjusted pH 7.3). The extracellular solution was composed of 140 mM NaCl, 5 mM KCl, 2 mM $CaCl_2$, 2 mM $MgCl_2$, 10 mM HEPES and 10 mM D-Glucose (300 mOsm; adjusted pH 7.4).

Upon establishment of stable whole-cell configuration, resting membrane potentials were calculated by 3-5 minutes recordings in I=0 mode. Action potentials (AP) were then evoked by 100 ms with 10pA current steps from -30 pA to +140 pA from -70 mV holding potentials. The evoked AP were analyzed using the Clampfit Module of the pCLAMP Software version 10.5

(Molecular devices) and GraphPad Prism. In Figures 3B-D and Figure S6, the rising time refers to the time elapsed between the stimulation and the AP peak; the decay time to the time between the AP peak and the end of the repolarization state; and the amplitude to voltage change in the rising phase of the AP.

ERK phosphorylation assay

KO#9 cells were seeded into six-well plates at a density of 3×10^5 cells per well in 2 mL of DMEM/F12 (1:1) supplemented 1% GlutaMAX (Gibco), 100 IU/mL Pen-Strep and 10% FBS. After 24 h, cells were co-transfected with a construct of a full length human *GRM5* (NM_001143831.2) (Sino Biological; #HG29734-ACG) and a *NCDN* construct containing the variant c.1297G>C, c.1433G>A, c.1492T>C, c.1955C>T or WT *NCDN*, respectively, using the jetPRIME transfection reagent (Polyplus). At 24 h post-transfection, cells were serum-starved overnight and at 48 h post-transfection, cells were washed with PBS and seeded in fresh starvation medium containing 100 µM (RS)-3,5-dihydroxyphenylglycine (DHPG) (R&D systems). Following stimulation for 5 min, medium was removed and cells were washed with PBS and then lysed in 100 µL cold RIPA buffer (Sigma), supplemented with complete, EDTA-free protease inhibitor cocktail (Roche). Protein concentration was measured by BCA protein assay (Pierce) and the phosphorylation of ERK1/2 was determined by western blotting as described above. Primary rabbit polyclonal antibodies against phosphorylated ERK1/2 (Thr202/Tyr204) and total ERK1/2 were used (Cell Signaling Technology). The results were analyzed by Image Studio version 5.2 (LI-COR). The intensity of each band was normalized with total ERK. The average of relative band intensity of four blots from independent experiments were analyzed.

Cell-type-specific expression of *NCDN* in fetal cortex

A previously published single-cell RNA sequencing dataset (UCSC Cell Browser (human cerebral cortex), https://cells.ucsc.edu/) was used to investigate specific gene expression in the developing human cerebral cortex.[8] We performed linear dimensional reduction (pca; npcs = 30) and t-stochastic neighbor embedding (tSNE) clustering (dims = 1:20, tsne.method = Rtsne) of 4261 cells using Seurat 3.[9] We defined three main cell clusters using cell markers from the original paper, namely 'radial glia' expressing the marker *VIM*, 'interneurons' expressing the marker *DLX1,* and 'excitatory neurons' expressing the marker *NEUROD6*.[8] Small clusters, including microglia and astrocytes, were annotated as 'Other' (Figure 3F). Within the excitatory neural cluster we similarly distinguished maturing excitatory neurons (mEN), expressing the marker *GRIN2B,* from the immature excitatory neurons (iEN), expressing the marker *NRP1*.[8]

Supplemental references

1. Sekiguchi, F., Tsurusaki, Y., Okamoto, N., Teik, K.W., Mizuno, S., Suzumura, H., Isidor, B., Ong, W.P., Haniffa, M., White, S.M., et al. (2019). Genetic abnormalities in a large cohort of Coffin–Siris syndrome patients. J. Hum. Genet. *64*, 1173–1186.

2. Wiel, L., Baakman, C., Gilissen, D., Veltman, J.A., Vriend, G., and Gilissen, C. (2019). MetaDome: Pathogenicity analysis of genetic variants through aggregation of homologous human protein domains. Hum. Mutat. *40*, 1030–1038.
3. Fatima, A., Schuster, J., Akram, T., Sobol, M., Hoeber, J., and Dahl, N. (2020). Generation of a human Neurochondrin deficient iPSC line KICRi002-A-3 using CRISPR/Cas9. Stem Cell Res. *44*, 101758.
4. Ronn, L.C.B., Ralets, I., Hartz, B.P., Bech, M., Berezin, A., Berezin, V., Moller, A., and Bock, E. (2000). A simple procedure for quantification of neurite outgrowth based on stereological principles. J Neurosci Methods. *10,* 25-32.
5. Shteinfer-Kuzmine, A., Argueti, S., Gupta, R., Shvil, N., Abu-Hamad, S., Gropper, Y., Hoeber, J., Magrì, A., Messina, A., Kozlova, E.N., et al. (2019). A VDAC1-Derived N-Terminal Peptide Inhibits Mutant SOD1-VDAC1 Interactions and Toxicity in the SOD1 Model of ALS. Front. Cell. Neurosci. *13*, 1–16.
6. Dmytriyeva, O., De Diego Ajenjo, A., Lundø, K., Hertz, H., Rasmussen, K.K., Christiansen, A.T., Klingelhofer, J., Nielsen, A.L., Hoeber, J., Kozlova, E., et al. (2020). Neurotrophic Effects of Vascular Endothelial Growth Factor B and Novel Mimetic Peptides on Neurons from the Central Nervous System. ACS Chem. Neurosci. *11*, 1270–1282.
7. Santillo, S., Moriello, A.S., and Maio, V. Di (2014). Electrophysiological variability in the SH-SY5Y cellular line. Gen. Physiol. Biophys. *33*, 121–129.
8. Nowakowski, T.J., Bhaduri, A., Pollen, A.A., Alvarado, B., Mostajo-Radji, M.A., Di Lullo, E., Haeussler, M., Sandoval-Espinosa, C., Liu, S.J., Velmeshev, D., et al. (2017). Spatiotemporal gene expression trajectories reveal developmental hierarchies of the human cortex. Science *358*, 1318–1323.
9. Butler, A., Hoffman, P., Smibert, P., Papalexi, E., and Satija, R. (2018). Integrating single-cell transcriptomic data across different conditions, technologies, and species. Nat. Biotechnol. *36*, 411–420.
